# Landscape impacts of 3D‐seismic surveys in the Arctic National Wildlife Refuge, Alaska

**DOI:** 10.1002/eap.2143

**Published:** 2020-05-26

**Authors:** Martha K. Raynolds, Janet C. Jorgenson, M. Torre Jorgenson, Mikhail Kanevskiy, Anna K. Liljedahl, Matthew Nolan, Matthew Sturm, Donald A. Walker

**Affiliations:** ^1^ Alaska Geobotany Center Institute of Arctic Biology & Department of Biology and Wildlife University of Alaska Fairbanks Alaska 99709 USA; ^2^ Arctic National Wildlife Refuge U.S. Fish and Wildlife Service Fairbanks Alaska 99701 USA; ^3^ Alaska Ecoscience Fairbanks Alaska 99709 USA; ^4^ Institute of Northern Engineering University of Alaska Fairbanks Fairbanks Alaska 99775 USA; ^5^ University of Alaska Fairbanks Fairbanks Alaska 99775 USA; ^6^ Woods Hole Research Center Falmouth Massachusetts 02540 USA; ^7^ Fairbanks Fodar Fairbanks Alaska 99708 USA; ^8^ Geophysical Institute University of Alaska Fairbanks Fairbanks Alaska 99775 USA

**Keywords:** 1002 Area, 3D seismic, Alaska, Arctic National Wildlife Refuge, cumulative impacts, hydrology, ice‐rich permafrost, oil and gas exploration, snow, tundra

## Abstract

Although three‐dimensional (3D) seismic surveys have improved the success rate of exploratory drilling for oil and gas, the impacts have received little scientific scrutiny, despite affecting more area than any other oil and gas activity. To aid policy‐makers and scientists, we reviewed studies of the landscape impacts of 3D‐seismic surveys in the Arctic. We analyzed a proposed 3D‐seismic program in northeast Alaska, in the northern Arctic National Wildlife Refuge, which includes a grid 63,000 km of seismic trails and additional camp‐move trails. Current regulations are not adequate to eliminate impacts from these activities. We address issues related to the high‐density of 3D trails compared to 2D methods, with larger crews, more camps, and more vehicles. We focus on consequences to the hilly landscapes, including microtopography, snow, vegetation, hydrology, active layers, and permafrost. Based on studies of 2D‐seismic trails created in 1984–1985 in the same area by similar types of vehicles, under similar regulations, approximately 122 km^2^ would likely sustain direct medium‐ to high‐level disturbance from the proposed exploration, with possibly expanded impacts through permafrost degradation and hydrological connectivity. Strong winds are common, and snow cover necessary to minimize impacts from vehicles is windblown and inadequate to protect much of the area. Studies of 2D‐seismic impacts have shown that moist vegetation types, which dominate the area, sustain longer‐lasting damage than wet or dry types, and that the heavy vehicles used for mobile camps caused the most damage. The permafrost is ice rich, which combined with the hilly topography, makes it especially susceptible to thermokarst and erosion triggered by winter vehicle traffic. The effects of climate warming will exacerbate the impacts of winter travel due to warmer permafrost and a shift of precipitation from snow to rain. The cumulative impacts of 3D‐seismic traffic in tundra areas need to be better assessed, together with the effects of climate change and the industrial development that would likely follow. Current data needs include studies of the impacts of 3D‐seismic exploration, better climate records for the Arctic National Wildlife Refuge, especially for wind and snow; and high‐resolution maps of topography, ground ice, hydrology, and vegetation.

## Introduction

This review is intended to help inform decision‐making and the permitting process involved in conducting 3D‐seismic surveys during oil and gas exploration in tundra regions throughout the Arctic. It draws attention to the sparse scientific information regarding the impacts of 3D‐seismic exploration, despite the fact that these programs are the largest single source of annual terrain impacts generated by oil and gas activities (Orians et al. 2003). Previous summaries of the impacts of seismic work focused mostly on the boreal forest in Canada (e.g., Dabros et al. 2018). Broader studies of the cumulative impacts of Arctic oil development focused on infrastructure and its effects on permafrost (Orians et al. 2003, Becker and Pollard 2016, McCarter et al. 2017, Vincent et al. 2017). Although this review is limited to landscape impacts, we also emphasize its broader relevance to the water, wildlife, and the people who depend on tundra resources for subsistence and recreation.

Seismic reflection exploration has been used for the last century to locate subsurface geological formations that might hold oil and gas reserves (Sheriff and Geldart 1995). The technique creates acoustic energy near the surface, either with an explosion or a vibrating vehicle (Vibroseis method), and then records the travel time of reflected waves to determine the depths of various strata. Historically, most seismic surveys were conducted along lines and interpreted in two dimensions (2D). The 2D lines were spaced kilometers apart: too far to effectively interpolate underground stratigraphy. Increased computing capability and improved methodologies have allowed geophysicists to collect more closely spaced lines of seismic data and to combine these lines into three‐dimensional (3D) models of the subsurface geology (Liner 2004).

In Arctic Alaska, seismic exploration is now only permitted on frozen ground with adequate snow cover, due to the significant impacts of summer travel on tundra (e.g., Rickard and Brown 1974). Studies of winter off‐road traffic show that despite efforts to reduce impacts, such as requiring minimum snow cover and freeze depth, impacts continue to occur, resulting in some areas with permanent changes to landscape and vegetation (Orians et al 2003, Bader 2006, Bureau of Land Management 2008, Jorgenson et al. 2010).

## The Arctic National Wildlife Refuge and the 1002 Area

**Fig. 1:**
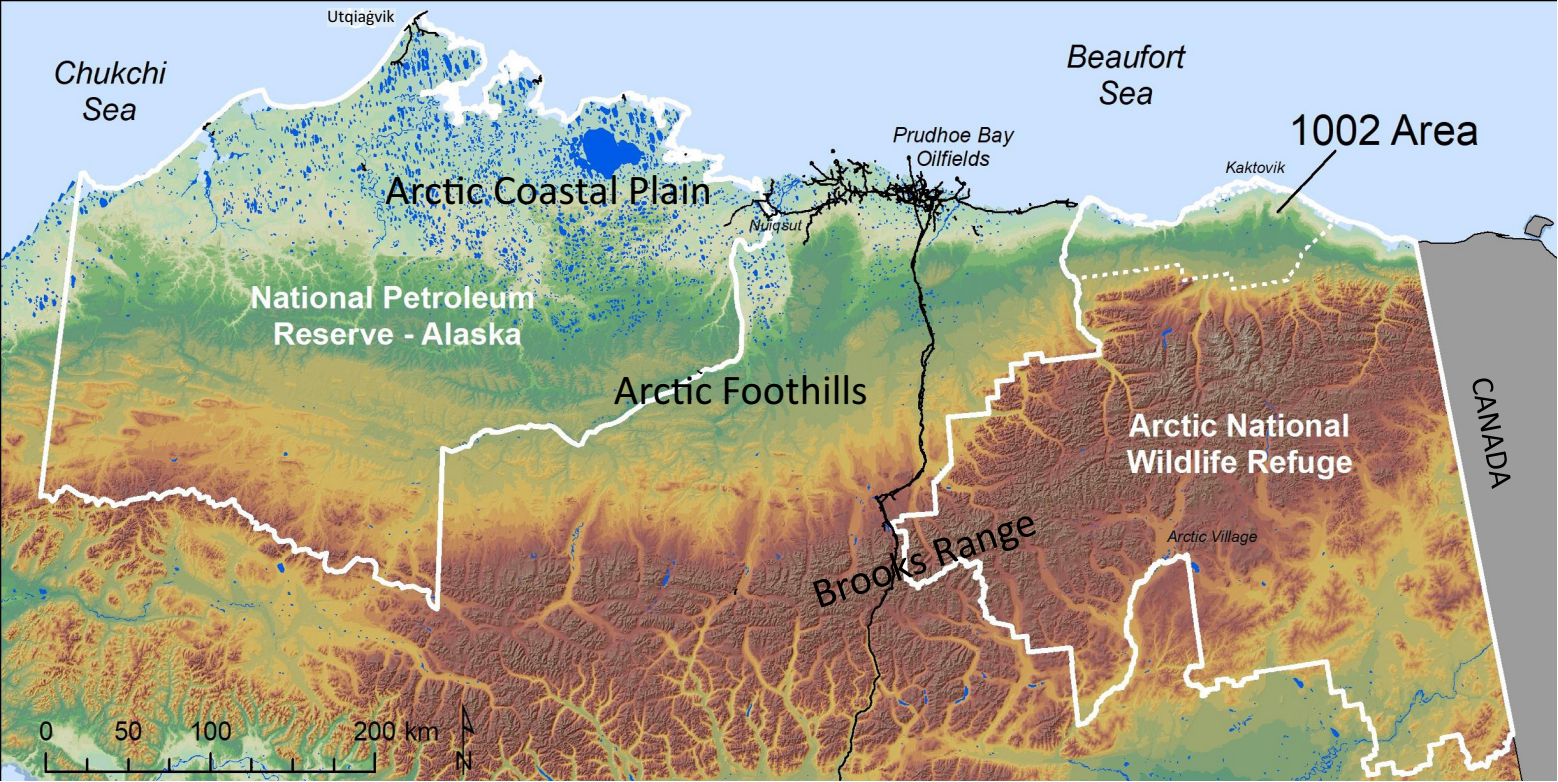
Topography and surface waters of Northern Alaska, USA, showing the National Petroleum Reserve–Alaska (NPR‐A) to the west, Prudhoe Bay oilfield roads, the Dalton Highway and Trans‐Alaska Pipeline extending south from Prudhoe Bay, and the Arctic National Wildlife Refuge (Arctic NWR) to the east (to the Canadian border). The 1002 Area within the Arctic NWR is shown with a dotted line.

Here we introduce the Arctic National Wildlife Refuge (Arctic NWR), as we use a proposed 3D‐seismic survey in this area to examine potential landscape impacts of 3D‐seismic exploration in the Arctic. The Arctic NWR was established in 1980 by the Alaska National Interest Lands Conservation Act (ANILCA), which expanded the Arctic National Wildlife Range (established in 1960). Section 1002 of ANILCA mandated studies of the natural resource potential of a 6,327 km^2^ area in the northern part of the Arctic NWR (referred to here as the 1002 Area) (Fig. 1), including the biological, geological, and oil and gas resources. As part of the studies of oil and gas resources, 2D‐seismic surveys were conducted in the 1002 Area during the winter/spring of 1984 and 1985 and the results reported in a U.S. Geological Survey Open File report (USGS 1998). The consequences of the trails created by the 2D surveys have been studied for decades by the U.S. Fish and Wildlife Service (USFWS; e.g., Jorgenson et al. 2010) and are described below under Landscape Impacts of Seismic Surveys in the 1002 Area.

**Fig. 2:**
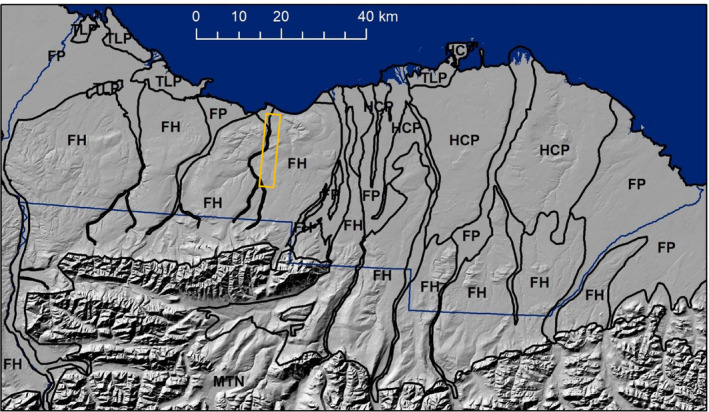
Primary terrain units of the 1002 Area (blue boundary) of the Arctic National Wildlife Refuge, overlain on a hill‐shaded terrain map. Unit boundaries from Walker et al. (1982). The area of snow mapping (Fig. 5) is shown in the orange rectangle. The terrain units within the 1002 Area in order of dominance are FH, foothills (45%); RF, river floodplains and deltas (25%); HCP, hilly coastal plains (22%); TLP, thaw‐lake plains (3%); and MTN, mountainous terrain (0.03%).

**Fig. 3:**
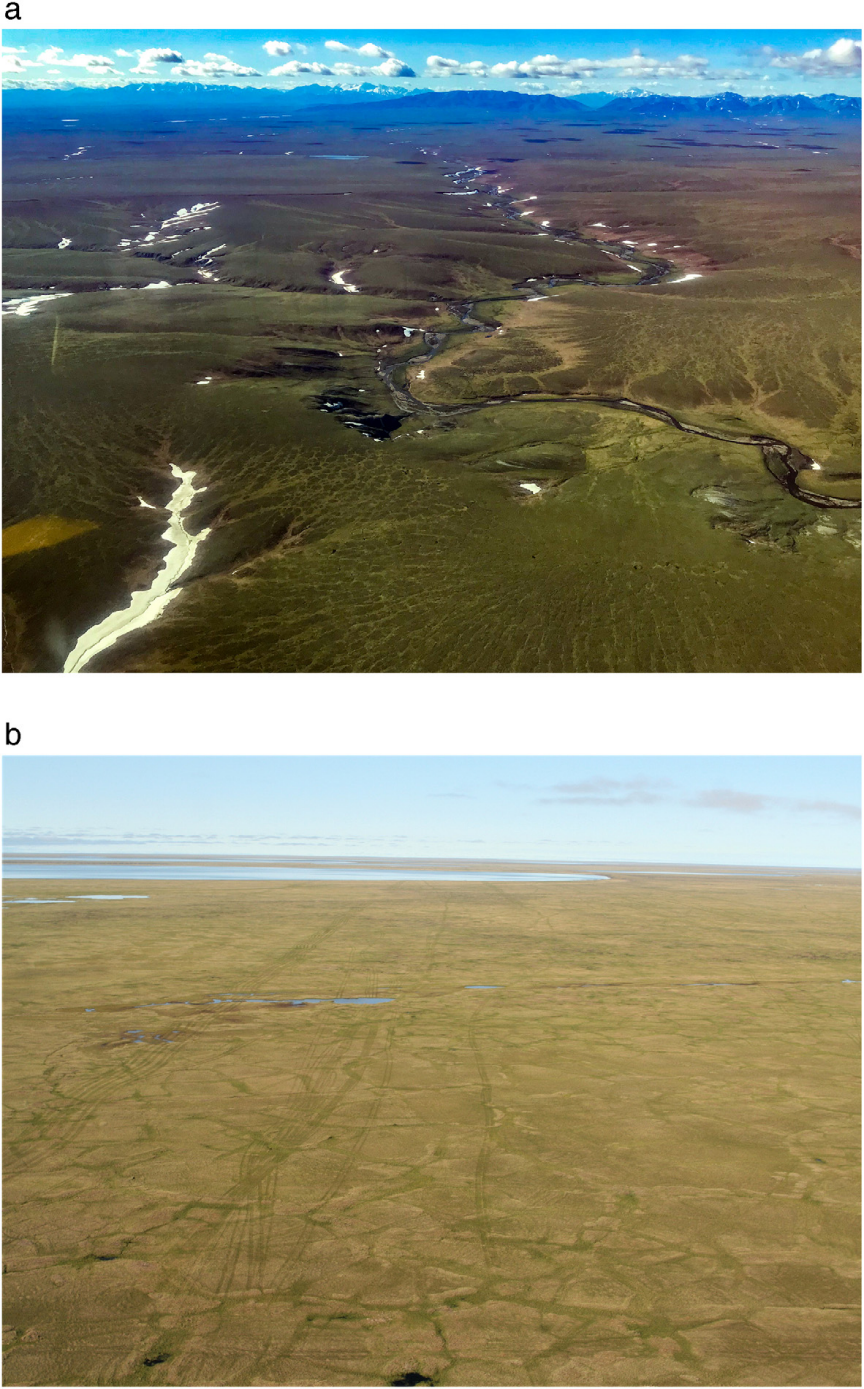
(a) Hilly terrain of the foothills portion (45%) of the 1002 Area of the Arctic National Wildlife Refuge. Snow regimes are highly variable, and hilly terrain would be difficult for seismic vehicles and camp trains to negotiate (photo: M. Nolan). (b) Flat coastal plain of the North Slope (Cape Halkett area, NPR‐A), where the majority of 3D‐seismic surveys on the North Slope have occurred (photo: M. T. Jorgenson).

The Arctic NWR is geologically and ecologically different from the rest of Arctic Alaska. The North Slope of Alaska extends northward from the Brooks Range mountains to the Beaufort Sea, varying in width from 300 km in the west to <50 km within the Arctic NWR. There, the Brooks Range is close to the Beaufort Sea, compressing the transition from the mountains to the ocean (Fig. 1). This creates steeper topographic gradients and stronger winds compared to the flatter, western North Slope. A map of the terrain types of the 1002 Area (Walker et al. 1982; Fig. 2) shows that it is dominated by foothills (45%; Fig. 3a) and hilly coastal plains (22%). River floodplains and deltas cover 25%, and flat thaw‐lake plains comprise about 3% of the 1002 Area (Fig. 3b) shows a thaw‐lake plain in the Prudhoe Bay area). This dissected terrain is not usually envisioned when the 1002 Area is referred to as a “coastal plain,” as in the BLM’s *Coastal Plain Oil and Gas Leasing Program Environmental Impact Statement* (Bureau of Land Management 2019). Mineral‐rich granite and limestone bedrock and glaciers of the Brooks Range mountains just south of the 1002 Area feed its numerous rivers and floodplains, and have carved the landscape into deep ravines and channels. The steep topographic gradients are reflected in the diverse geology, soils, snow regimes, hydrology, and vegetation, which form a mosaic of habitats supporting the high biological diversity of the region.

The Arctic NWR is poorly studied compared to the central North Slope. Most oil production on the Alaska North Slope is centered around Prudhoe Bay and westward into the National Petroleum Reserve–Alaska (NPR‐A). Most scientific research in northern Alaska has been concentrated along the Dalton Highway, in the Prudhoe Bay oilfields, and in the Utqiagvik (Barrow) region; all areas far west of the Arctic NWR and with landscapes quite different from the hilly, highly dissected terrain of the 1002 Area.

In addition to the landscape impacts of seismic trails discussed in this paper, there would also be impacts to wildlife. Caribou are the most abundant large mammals in the 1002 Area and are an important subsistence and cultural resource for Gwich'in, Iñupiaq, and Inuvialuit who hunt the Porcupine Caribou Herd (PCH) and Central Arctic Herd (CAH) in Alaska and Canada (Bureau of Land Management 2018). The caribou use the 1002 Area for calving in the spring and for insect relief in the summer, and their habitat is permanently reduced by avoidance of oil infrastructure (Johnson et al. 2019). Polar bears use the coastal zone for fall feeding and for maternal denning. As sea ice has become thinner and more prone to fragmentation, there has been a landward shift in the distribution of polar bear dens (Durner et al. 2006). Denning density is particularly high in the 1002 Area and the adjacent Ivvavik National Park in Canada, partly due to the hilly terrain, which provides deep snow drifts for denning sites. Most birds in the area are migratory, with 157 species recorded in the area. About one‐half of those are confirmed breeders and/or permanent residents, and others use the area during staging or migration (Bureau of Land Management 2018). Many shorebirds preferentially use the river deltas in the 1002 Area that are fed by nearby Brooks Range glaciers and host freshwater invertebrates (Nolan et al. 2011). The area also provides habitat to fish and other aquatic species in the lagoons and nearshore brackish waters of the Beaufort Sea; and the rivers, streams, and springs flowing north from the Brooks Range (Bureau of Land Management 2018). The World Wildlife Fund recognized this region as having one of the most diverse examples of Arctic tundra in the circumpolar Arctic (World Wildlife Fund 2007).

## 3D‐Seismic Plan for the 1002 Area

In December 2017, the U.S. Congress passed the “Tax Act” (Public Law 115‐97), which included Section 20001, directing the Secretary of the Interior to develop an oil‐and‐gas leasing and development program for the 1002 Area. In anticipation of that leasing, SAExploration Inc., a seismic exploration company, the Arctic Slope Regional Corporation, and Kaktovik Iñupiat Corporation submitted a plan in July 2018 to conduct 3D‐seismic exploration over the entire 1002 Area (SAExploration 2018). The U.S. Bureau of Land Management (BLM) was tasked with evaluating the potential impacts of the activity.

BLM stated that they believed there would be “no significant impact” from SAExploration’s plans (Ruskin 27 July 2018) and therefore determined there was no need for a full environmental impact statement (EIS) under the National Environmental Policy Act (NEPA). The BLM determined that an environmental assessment (EA), a less detailed document, would be sufficient to evaluate and permit the seismic exploration activities (Ruskin 27 July 2018). The EA was not released, and in February 2019, BLM paused the seismic NEPA evaluation process (Fountain 7 Feb. 2019). In December 2018, BLM released a Draft EIS for oil and gas leasing in the 1002 Area that specifically did not address the 3D‐seismic exploration (Bureau of Land Management 2018). The Final EIS, released in September 2019, included some mention of seismic exploration, but no evaluation of its impacts, nor a summary of the area that would likely be affected (Bureau of Land Management 2019). At the time of this paper, the process by which the seismic exploration work would be evaluated remains uncertain.

The SAExploration plan called for 200‐m spacing between seismic lines (SAExploration 2018). This would create a grid of more than 63,000 km of seismic trails in the 1002 Area (see Appendix S1: Fig. S1 for an impression of the density of the proposed seismic lines). The width of the resulting trails created by numerous vehicles was assumed to be approximately 10 m based on the width we measured on aerial photos of recent 3D‐seismic exploration adjacent to the 1002 Area by the same company proposing the exploration in the Arctic NWR. This means the seismic trails would directly affect approximately 630 km^2^, equivalent to approximately 10% of the 1002 Area.

As proposed, two teams would conduct seismic surveys simultaneously (see Appendix S1: Table S1 for list of vehicles per team). These teams would be supported by two mobile camps, with trailers containing portable housing units, kitchens, and other facilities for approximately 160 workers each (SAExploration 2018). The camp trailers would be mounted on steel‐runner sleds and moved every 2–3 days to keep within approximately 5 km of the current survey area, as the crews progressed across the tundra. Each camp would be moved by 8–10 strings of five to eight trailers forming “cat trains” pulled by tractors or bulldozers. Rubber‐tracked agricultural tractors would pull the cat trains when possible, and steel‐tracked D7‐Caterpillar bulldozers would be needed to pull the heaviest trains and would be used in steep terrain or snow accumulation areas, both of which are common in the 1002 Area. SAExploration estimated that there would be 40‐50 different camp locations within the 1002 Area.

While there have been improvements in reducing the ground pressure of some vehicles, fleet sizes for the proposed exploration are more than double those of the previous surveys in the 1002 Area in 1984–1985, and some vehicles are heavier. Table 1 summarizes vehicles used historically and those proposed for the 1002‐Area survey (SAExploration 2018). Impacts to the tundra terrain caused by the proposed 3D‐seismic exploration would include (1) grids of seismic trails, totaling approximately 63,000 km, created by tracked vibrator units (Appendix S1: Fig. S2), tracked receiver vehicles, geophone carriers, and tracked personnel carriers (Appendix S1: Fig. S3); (2) camp‐move trails, minimally estimated at 580 km, created by 8–10 strings of five to eight camp and fuel sleds (Appendix S1: Figs. S4, S5) pulled by tractors (Appendix S1: Fig. S6) or bulldozers (Appendix S1: Fig. S7) and a variety of other vehicles such as Rolligons and front‐end loaders (Appendix S1: Figs. S8, S9); (3) impacted areas at 40–50 camp locations (see Appendix S1: Fig. S1, bottom, for satellite view of seismic camps in the NPR‐A); (4) airstrips to support the camps (SAExploration states these would be within 8 km of every camp, so approximately 25 airstrips); (5) trails to and from the camps to the seismic work areas; and (6) trails made by fuel haulers (Appendix S1: Fig. S10) traveling from the distant road system to camps.

**Table 1:** Seismic survey vehicles, ground pressure per vehicle, and number of units for one survey crew during previous seismic surveys in the North Slope of Alaska from 1984–1985 to 2017.

		1984	1985	1996	1999	2000	2001	2017	2020	
Vehicle type	Ground pressure, 1000 pascals (psi)	ANWR 2D	ANWR 2D	Colville 3D	NPR‐A 3D	W of Colville 3D	E of Colville 3D	NPR‐A GMT 3D	Proposed ANWR 3D	Photo (see Supp. Info.)
Vibrators and other vehicles for line work and crew transport										
Vibrators	31–103 (4.5–15)	0†	6	10	10	10	10	12	12	S2
Other‡	7–97 (1–14)	23†	15	6	15	15	24	36	41	S3, S8
Camp vehicles										
D7 Caterpillar tractor	71 (10.3)	6	6	6	4	4	3	4	2	S7
Challenger or Case/Steiger tractor	31 (4.5)	0	0	0	0	0	1	10	9	S6
Camp sled on skis	41 (6)	14	12	~20	~25	24	33	38	~50	S4, S5
Caterpillar 977 loader	69 (10)	0	0	0	2	2	1	1	1	S9
Nodwell with crane	21 (3)	1	1	0	0	0	0	0	0	No photo
Fuel tanks on vehicles or sleds	41–55 (6–8)	3	4	5	7	6	7	8	11	S10
Total no. units, approximate		47	44	~47	~63	61	79	109	~127	

Notes

The last column shows the proposed vehicles for one of the two crews proposed for the survey in the 1002 Area (SAExploration 2018). We note the general location of the survey, and whether it was 2D or 3D. See Appendix S1: Figs. S2–S10 for photos of vehicles. Data were summarized from Jorgenson et al. (2003), from the BLM Greater Moose’s Tooth EA (Bureau of Land Management 2016), and the SAExploration Plan of Operations for ANWR (see Appendix S1: Table S1; SAExploration 2018).

† Drilled shothole technique used in 1984 instead of vibrators. Vehicles included nine drillers (19,000 pa) and one dynamite magazine (15,000 pa).

‡ “Other” includes vehicles used for recorders, geophone deployment, crew transport, mainly 7,000–35,000 pa but a few up to 97,000 in the 1990s.

During the 1984–1985 2D surveys in the 1002 Area, the trails created by moving the camps created the most damage to the tundra and will be of special concern for any proposed 3D surveys. Camps and camp‐move trails are made by vehicles with higher ground pressure than those on the seismic lines, and therefore cause more initial damage and have slower recovery (Jorgenson et al. 2010, Bureau of Land Management 2012). In 1989, five years after disturbance, 64% of camp‐move trails were still disturbed compared to 32% of the seismic trails, and 41% of the camp‐move trails still had medium‐ and high‐level disturbance. Measurable disturbance remained on 10% of camp‐move trails in 2009 (Jorgenson et al. 2010) and 5% in 2018, 33–34 yr after the trails were made (Jorgenson 2018).

The camp‐move trails associated with the 1984–1985 surveys were equal in length to the seismic trails, about 2,000 km, and generally wider than seismic lines, from 4 to >50 m width (Jorgenson et al. 2010). An average width of 20 m gives an estimate of 11.6 km^2^ of tundra disturbed by the proposed camp‐move trails (Table 2). The 3D‐seismic survey would have proportionately fewer kilometers of camp‐move trails than the 2D survey because the seismic grid would be so closely spaced, but the 580 km of camp‐move trails stated in the SAExploration plan should be considered a minimum. The actual length of camp trails would probably be longer because trails would have to follow nonlinear routes to avoid steep slopes and areas with inadequate snow cover, and to access suitable campsites and airplane landing strips.

**Table 2:** Estimated length and area of seismic trails, camp‐move trails, and total area of trails in different vegetation types for proposed 3D‐seismic exploration of the 1002 Area of the Arctic National Wildlife Refuge.

Vegetation type	Seismic trails		Camp‐move trails		Total area of trails (km^2^)	Area with initial low‐level disturbance (km^2^)	Area with initial medium‐ to high‐level disturbance (km^2^)
	Length (km)	Area (km^2^)	Length (km)	Area (km^2^)			
Moist sedge/willow tundra	23,078	230.8	209	4.2	235.0	202.6	32.32
Moist tussock tundra	18,478	184.8	97	1.9	186.7	124.9	61.81
Wet sedge tundra	9,654	96.5	101	2.0	98.6	98.5	0.04
Moist sedge–*Dryas* tundra	6,017	60.2	42	0.8	61.0	43.7	17.31
Moist dwarf‐shrub tundra	2,781	27.8	43	0.9	28.7	20.4	8.26
Riparian low shrubs	909	9.1	8	0.2	9.3	8.3	0.96
Dry *Dryas* river terrace	214	2.1	0	0.0	2.1	1.1	1.08
Partially vegetated	1,043	10.4	51	1.0	11.4	11.4	0
Water	963	9.6	27	0.5	10.2	10.2	0
Total	63,270	632.7	580	11.6	644.3	511.0	121.8

Notes

The total length of trails are from SAExploration (2018). The estimated length of seismic trails and camp‐move trails in each vegetation type and the amount of initial disturbance were based on the amount of each vegetation type traversed by 1984–1985 trails and the amount of initial disturbance caused by each type of trail in each vegetation type (Raynolds and Felix 1989). All values are estimates.

## Landscape Impacts of Seismic Surveys in the 1002 Area

Most of the known effects of seismic exploration to Alaska tundra vegetation come from USFWS studies of 2D‐seismic trails that were made during and after the 1984–1985 seismic surveys in the 1002 Area. USFWS personnel accompanied the seismic teams and established long‐term study plots to observe the snow conditions and impacts, and followed up with periodic observations of recovery that continued through 2018 (Jorgenson 2018). Results of 25 years of the study were reported previously in this journal (Jorgenson et al. 2010). These studies are relevant to the proposed 3D‐exploration plan because the main cause of disturbance would be similar vehicle traffic. However, the areal extent of the impacts would likely be over 30 times those generated by the 1984–1985 campaign (SAExploration 2018), and many vehicles would be heavier than those used in the 1984–1985 surveys (Table 1).

To discuss the impacts of seismic exploration, we follow the three‐layer permafrost model described by Vincent et al. (2017). The top layer is directly affected by the atmosphere, and includes snow and vegetation, both of which buffer the underlying soil from the direct effects of air temperature and precipitation. The second layer is the active layer, that part of the soil that thaws annually, and below that is permafrost (the third layer), which underlies all of the 1002 Area. Seismic exploration would directly affect the top layer, leading to indirect effects on the second and third layers. There is a complex “transfer function” between a vehicle and the tundra mediated by the snow and vegetation, where the mechanical properties and layering of the materials are critical in determining how the load transfers. These immediate effects are poorly understood, but below we document what is known about both the initial and long‐term effects of winter travel on tundra.

### Snow

The relationship between snow characteristics and disturbance from the 1984–1985 seismic surveys was analyzed for the two most common vegetation types, tussock tundra and moist sedge–willow tundra (Felix and Raynolds 1989*b*). Snow depths were usually less than 30 cm and did not provide complete protection from vehicle damage (Appendix S1: Figs. S11, S12). Medium‐level (long‐lasting) disturbance (see Appendix S1: Table S4 for disturbance rating criteria) occurred at snow depths up to 25 cm in tussock tundra and up to 35 cm in moist sedge–willow tundra (Felix and Raynolds 1989*b*). Disturbance was less where snow was deeper, particularly depths in excess of 25 cm. The thickness of a wind‐slab layer (a harder, denser layer on top of softer snow) was a better predictor of the degree of vegetation protection than total snow depth. A wind‐slab depth of 20 cm above a soft depth‐hoar layer (a very loose layer consisting of large, fragile crystals that forms at the base of a cold snowpack) appeared to be sufficient to prevent most disturbances from seismic vehicles, but not from the heavier camp‐move vehicles (Felix and Raynolds 1989*a*). The camp‐move trails from the 2D‐seismic exploration, especially the 1985 trails, followed drainages to take advantage of deeper snow. However, this type of travel may be restricted in the future, because these areas of deeper snow are also used by female polar bears for winter denning and birthing (Bureau of Land Management 2018).

To reach the 1002 Area from the road system, vehicles would have to cross both the Sagavanirktok and Canning Rivers, braided rivers with large deltas. These deltas are vulnerable to large icing events (Shur et al. 2016). In 2015, snow compaction caused by seismic trails and the resulting reduced insulation above the water table were implicated in causing icing, which resulted in extensive flooding and erosion of the Dalton Highway to Prudhoe Bay (Shur et al. 2016).

The land between the road system and the Arctic NWR is a mix of Alaska State and U.S. Federal lands, managed by different agencies that enforce different criteria for determining adequate snow cover. On State lands, the Alaska Department of Natural Resources (ADNR) monitors snow depth and soil temperature at a series of sites along the road system to determine the dates to open and close the tundra to winter vehicle travel. Minimum conditions are based on studies conducted in 2003 and 2004 (Bader 2006). To begin the open tundra travel season, ADNR requires >15 cm (6 in) of snow for the coastal plain ecoregion and 23 cm (9 in) for the foothills, where tussock‐forming sedges create greater microrelief. The density of the snow is considered, recognizing that low density snow provides less protection than higher density snow (Felix and Raynolds 1989*a*). Soil temperatures in all locations have to be ≤ −5°C at 0.3 m depth (SAExploration 2018). Tundra travel is closed in the spring when the snowpack starts to deteriorate due to warm temperatures and increased sunlight. ADNR has subdivided the coastal plain into eastern and western sections, and the foothills into upper and lower sections, with different monitoring measurements and opening/closing dates regulated for each area (Northern Oil & Gas Team, ADNR, *personal communication*).

On federal lands, BLM regulates winter vehicle traffic, and has recently stopped using the ADNR system and instead uses a “performance‐based” system whereby the operator decides when there is enough snow (Bureau of Land Management 2013). The effectiveness of the performance‐based system in preventing tundra damage has not been studied in any rigorous way. During the 1984–1985 2D‐seismic surveys in the 1002 Area, USFWS regulations required a minimum average snow depth of 15 cm (Felix and Raynolds 1989*b*). USFWS monitors travelled with the seismic crews, measuring snow depths and recording vehicle impacts to vegetation and soils. For the currently proposed 3D‐seismic exploration, it is unclear where and how often snow monitoring would occur, who would do it, who would review the data, and who would make the decisions as to when to allow or halt winter travel in the 1002 Area.

**Fig. 4:**
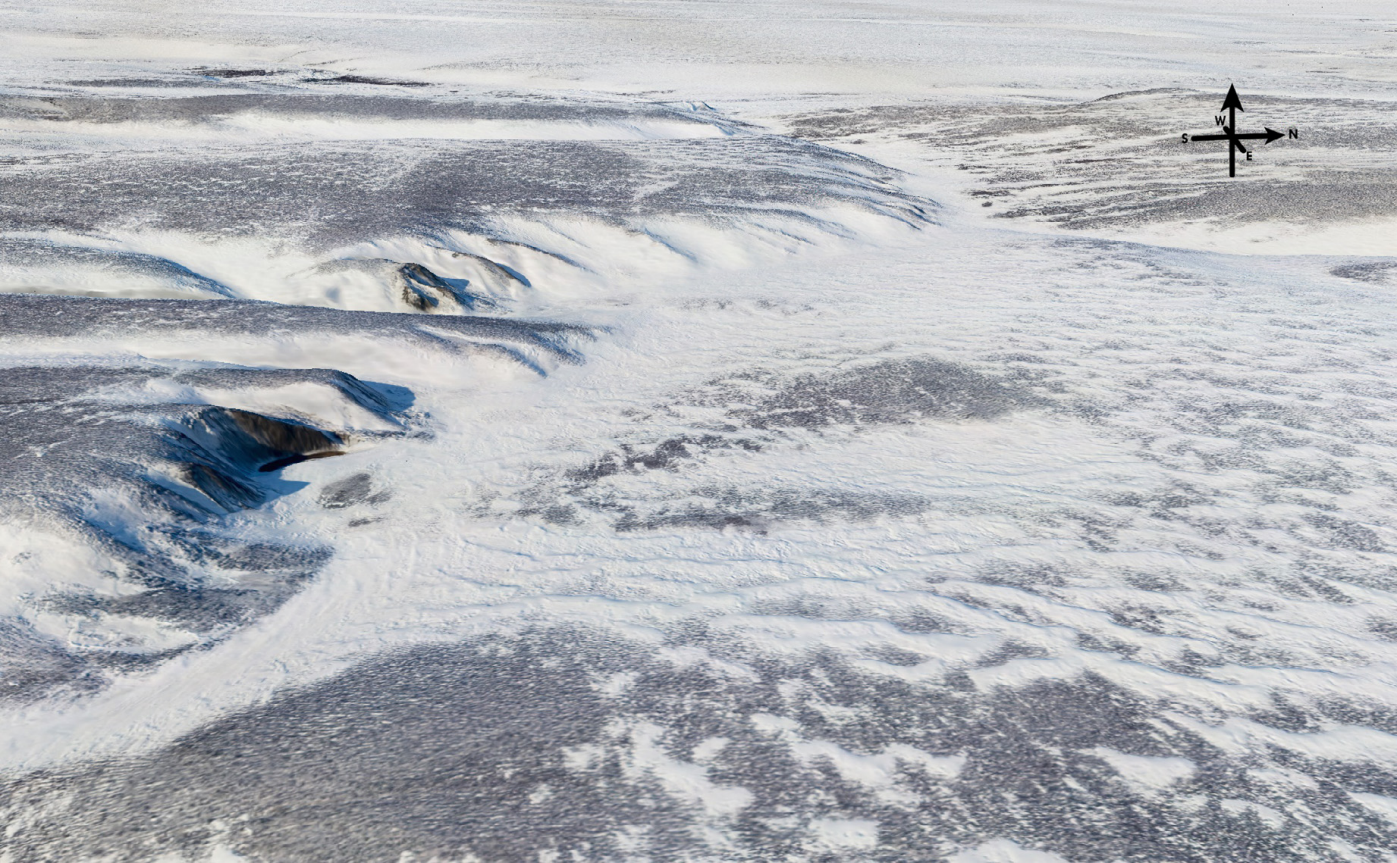
Patchy snow cover on foothills terrain in the 1002 Area, Arctic National Wildlife Refuge, March 2019 (photo: M. Nolan).

**Fig. 5:**
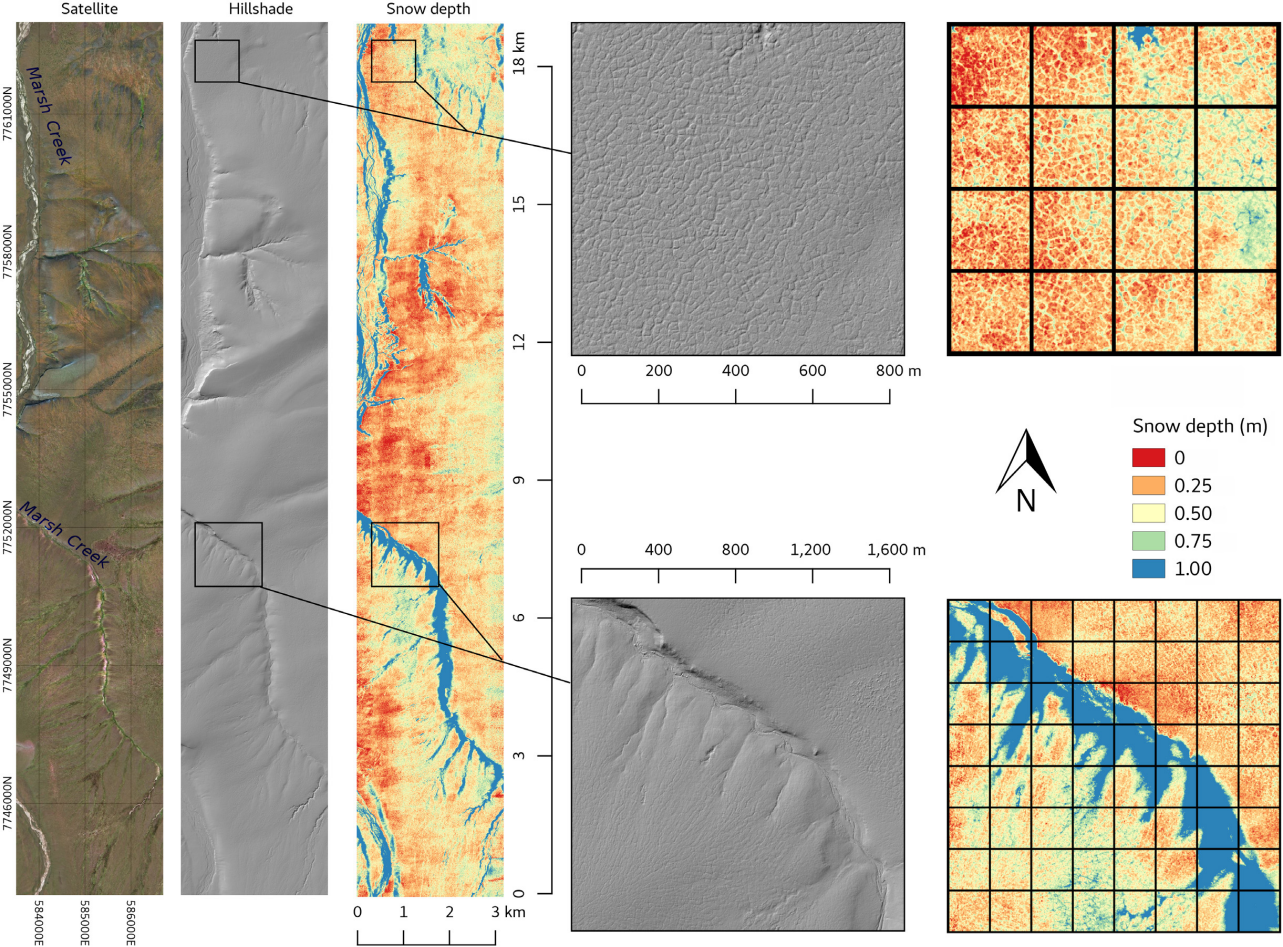
From left to right, summer terrain, winter aerial photograph, and snow depth calculated from airborne elevation surveys in April 2018 of a 3 × 19 km area centered on Marsh Creek, Arctic National Wildlife Refuge. The method is described in Nolan et al. (2015). The location of this imagery is shown by the orange rectangle in Fig. 2. The grid on the two inset maps on the right shows 200 × 200 m spacing as proposed for the seismic exploration (SAExploration 2018). The width of the lines approximating the average 10 m width of the seismic trails.

There has been no comprehensive study of snow cover in the 1002 Area, such as continuous and/or multi‐year or landscape‐scale snow accumulation measurements. However, observations from the 1984–1985 2D‐seismic surveys and more recent aerial observations in 2018 and 2019 indicate that snow cover over most of the area is generally thin (<50 cm), and over much of the 1002 Area tussock tops are bare throughout the winter due to snow redistribution (Fig. 4). Large snowdrifts 2‐ to 5‐m deep occur adjacent to scour areas along the many incised stream and river valleys (Figs. 4, 5). The scoured areas are the sources of the snow that forms the drifts (Nolan et al. 2015). Snow monitoring in the region has been sparse, has suffered from changing station locations, and contains large data gaps. A 41‐yr (1948–1989) period of continuous measurements from the Beaufort Sea coast at Kaktovik, north of the 1002 Area, showed annual maximum snow depths varying from 20 to 120 cm in this flat, coastal location (Appendix S1: Fig. S13). This sixfold variation reflects both local landscape variations in snow cover and errors, highlighting the difficulty of measuring snow with point samples in this windy tundra region (Black 1954, Benson 1982).

The only additional snow information for the 1002 Area comes from three permafrost monitoring stations operated by the USGS (Urban and Clow 2013), where wind‐speed and one‐point snow‐depth data are collected by autonomous instruments. Unfortunately, no overlap exists between the older weather records and these newer data (since 2000–2003), hence identifying current trends in snow depth for the 1002 Area is not possible. Additionally, while sonic depth‐sounder measurements like those operated by the USGS offer an inexpensive way to monitor snow depth autonomously, the unshielded gauges are notoriously inaccurate in windy snow regions (e.g., Sevruk et al. 2009). They also only record snow depth, not snow‐water equivalent, and therefore do not provide any estimate of snow density, which is closely related to the protective characteristics of snow (Felix and Raynolds 1989*b*). The USGS measurements show that average monthly snow depths from October through April, 2000–2013 were 21 cm at the coast, 15 cm on the flatter eastern portion of the 1002 Area, and 20 cm in the hilly western portion (Urban and Clow 2013). A monthly average depth of 15 cm (required by the State of Alaska for travel on the North Slope coastal plain) was not reached at the coastal site in 1 of the 10 yr measured, and at the eastern site in 3 of 14 yr. The 23‐cm snow depth required by the State of Alaska for travel in the foothills was not reached at the hilly western site in 6 of the 13 yr measured (2001–2013; Urban and Clow 2013).

A photogrammetrically derived snow‐depth map produced by the authors for an area in the western part of the 1002 Area demonstrates the range of snow depths typically encountered as a result of snow redistribution by wind (Fig. 5). This map of a 3 × 19 km area along Marsh Creek was made by subtracting a digital elevation model (DEM) of the summer ground surface from the late winter snow surface, following methods described by Nolan et al. (2015). We validated the map using ground measurements of snow depths. This example is characteristic of a larger study, still in analysis, using the same methods to measure snow depth over one‐quarter of the 1002 Area in March–April 2019.

Both the 2018 and 2019 surveys showed extensive areas of very low snow with exposed tussock tops. The high‐resolution inset maps show the pattern of deep snow (>1 m depth, blue color) in creek channels, and shallow snow (0–50 cm, red to yellow colors) on the creek bluffs (Fig. 5). High‐centered ice‐wedge polygons were abundant along the creek bluffs and extended more than 3 km upwind on the east side of the creek with no snow to shallow snow (<25 cm, red to orange colors) on the raised polygon centers (approximately 10–30 m width) and deeper snow (to 50 cm, yellow colors) in the polygon troughs (approximately 0.5 to >2 m width). Drifts in excess of 1 m deep (blue) were found immediately adjacent to scoured areas where the snow depth was less than 25 cm. These areas of thin and thick snow are conjugates, produced by wind removing snow from large areas of tundra and depositing it in much smaller topographic collection zones. Grids on the inset maps in Fig. 5, showing a spacing of 200 m between 10‐m wide seismic lines, demonstrates that completely avoiding low‐snow (<25 cm) areas with such a grid spacing would not be possible.

While there is no in‐depth analysis of winter wind speeds in the 1002 Area, data indicate that blizzard winds are stronger in this eastern part of the North Slope than farther west (Schwerdtfeger 1973, ASOS 2020). There is little snow cover over large parts of the 1002 Area for long periods during the winter, due to strong winter winds that relocate the snow into depressions and cause snow loss through sublimation. The direction and causes of the winds vary depending on the location (Zhang et al. 2016). Downslope southerly katabatic winds are common in north‐south valleys in and near the mountains, and strong easterly “mountain barrier jets” occur over of much of the foothills and coastal areas (Schwerdtfeger 1973, Kozo 1980). We lack comprehensive records of where scour and drift occur, and have little information on how often excessive scour takes place in winter. Based on the authors’ experience, visual observations, and work in progress, the 1002 Area is more scoured and drifted than other parts of the North Slope.

**Fig. 6:**
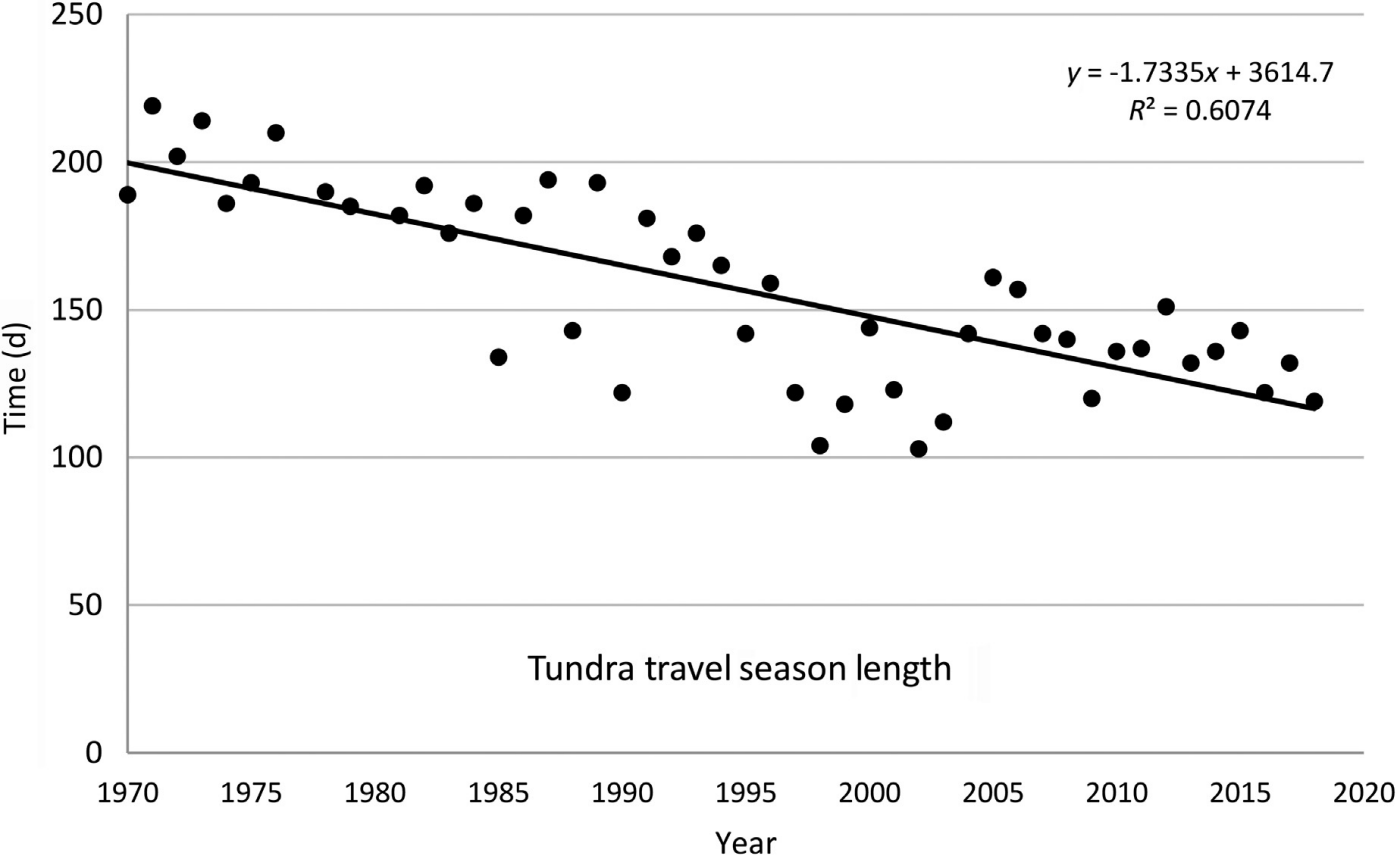
Number of days the Alaska Department of Natural Resources (ADNR) permitted winter tundra travel on the North Slope of Alaska. The year on the *x*‐axis denotes the spring season, with many seasons in the earlier part of the record opening in October or November of the preceding year, and many in the later part of the record opening in January of the year. Data are from a handout prepared by the ADNR Division of Mining, Land, & Water, Northern Regional Office, updated June 2018.

We also do not know how snow characteristics may be affected by the rapidly warming climate. Well‐documented climate warming in northern Alaska has caused later freeze‐up in fall and earlier snowmelt in spring, resulting in shortened permitted tundra‐travel seasons, from about 200 d in the 1970s to less than 120 d in the 2000s (Fig. 6; Hinzman et al. 2005, Bader 2006). The winter travel season in the foothills of the central North Slope has dropped below 100 d, and snow cover did not reach adequate depth (23 cm) for ADNR to open for travel there in 3 of the last 16 yr. Neither the upper nor lower foothills had enough snow to be opened during the winter of 2018–2019, the winter when SAExploration intended to start seismic surveys in the 1002 Area (Northern Oil & Gas Team, ADNR, *personal communication*). Some recent studies have suggested that with the reduction in Arctic sea ice and delayed freeze‐up in the fall, there should be an increase in October–December precipitation (Higgins and Cassano 2011, Carne 2017, Cai et al. 2018), but other predictions are that the increased precipitation will fall mainly as rain (Carne 2017), and that there will be an increase in winter rain‐on‐snow events (Bieniek et al. 2018), as well as changes in the direction and velocity of winds (Stegall and Zhang 2012).

### Vegetation

The vegetation of the 1002 Area has been described based on ground data and mapped using satellite data (Jorgenson et al. 1994). The tundra has a nearly continuous cover of plants <0.5 m tall, consisting mainly of sedges, dwarf shrubs, and mosses. The substrates of the different Arctic plant communities vary in their soil moisture, soil texture, amount of snow, depth of the active layer, and amount of ground ice (Walker et al. 2018). The plant growth forms and substrate characteristics affect the communities’ sensitivity to damage by winter vehicle traffic. Table 3 lists the common vegetation types in the 1002 Area and their relative sensitivity (Jorgenson et al. 2010). Appendix S1: Table S2 includes details on species composition, soil, and permafrost characteristics of the different plant communities.

**Table 3:** Common vegetation types, their proportion in the 1002 Area of the Arctic National Wildlife Refuge based on the vegetation types crossed by the 1984–1985 2D‐seismic surveys (Raynolds and Felix 1989), and their likelihood of long‐term disturbance (Jorgenson et al. 2010).

Vegetation type	Percent of 1002 Area	Likelihood of long‐term disturbance
Moist sedge–willow tundra	37	high
Moist tussock tundra	29	medium
Wet sedge tundra	15	low
Moist sedge–*Dryas* tundra	10	high
Moist dwarf‐shrub tundra	4	medium
Riparian low shrub	1	low
Dry *Dryas* river terrace	<1	medium
Partially vegetated	2	low
Water and aquatic vegetation	2	low

### 2D‐seismic impacts to vegetation

Studies of seismic impacts to vegetation were conducted by the USFWS after the 1984–1985 2D‐seismic exploration of the 1002 Area. A sample of 200 randomly selected permanent plots on trails, visited repeatedly over the following 33 yr, were used to quantify the percent of trails disturbed and recovery over time (Raynolds and Felix 1989, Jorgenson et al. 2015*a*, Jorgenson 2018). Other permanent plots were established to track recovery of plant cover by species in each vegetation type and at each level of initial disturbance (100 plots; Felix and Raynolds 1989*a*, Felix et al. 1992, Jorgenson et al. 2010, 2019).

#### Initial disturbance

Each plot in the USFWS study was rated for six disturbance factors including destruction of vegetation, exposed soil, and compression in the track. A summary disturbance rating (none, low‐level, medium‐level, or high‐level) was assigned to each plot (Appendix S1: Table S3). All percentages given in this section are from the random sample.

Trails were easily visible the first summer after the 2D‐seismic exploration and over 90% of the trails showed some form of disturbance. Three‐quarters of the trails had none to low levels of initial disturbance, and one quarter had medium‐ to high‐level disturbance. Disturbance varied greatly in relationship to snow cover and permafrost conditions, site moisture, microtopography, and vegetation characteristics (Jorgenson et al. 2010). Moist tundra (81% of the 1002 Area), including tussock tundra, was susceptible to damage because of the microtopographic relief, which is commonly 50 cm and can be up to 1 m. About one‐half of the plots in tussock tundra, shrub tundra, and *Dryas* terraces had medium‐ or high‐level initial disturbance in 1985, while this level of disturbance occurred in one‐third of sedge–*Dryas* tundra and moist sedge–willow tundra. Moist and dry vegetation types were most disturbed initially because of the abundance of easily damaged evergreen shrubs (Jorgenson et al. 2010). Almost 60% of riparian‐shrubland plots were initially impacted, despite generally deep snow on these plots, because of the much taller plant canopies (Raynolds and Felix 1989). Medium to high‐level disturbance occurred in less than 10% of the wet sedge tundra plots. Trails on bare or sparsely vegetated riverine gravels caused little or no disturbance.

The initial visibility of the trails was caused by vehicle compression of the vegetation and moss mat by about 20 cm. This led to increased moisture and organic matter decomposition, resulting in a vegetation greening response within the trails in the early years. Studies at Prudhoe Bay (Walker 1985), Toolik Lake (Chapin et al. 1979), Utqiagvik (Barrow) (Zona et al. 2011), and elsewhere (Ohlson and Dahlberg 1991) have shown that variations in microtopography account for much of the variation in biological diversity and ecosystem function of tundra landscapes. Compressing the tundra eliminates much of this microtopographic variability, which is important to the distribution of numerous plants and fungi, insects, small mammals, and birds.

#### Long‐term recovery

Visibility of the trails from the air decreased over the first five years, and trails with initial low‐level disturbance generally recovered well over the first decade. Many trails appeared brown due to dense dead sedge leaves and a reduction in shrubs (Appendix S1: Fig. S14). Measurable disturbance (Appendix S1: Table S4), such as changes in species cover, remained on 5% of trails in 2009 and 3% in 2018, 25 and 33 yr after disturbance, respectively (Jorgenson et al. 2010, Jorgenson 2018). Recovery of trails with medium‐ or high‐level disturbance took longer (Appendix S1: Fig. S15). One‐half of the trails with medium‐ to high‐level disturbance were still disturbed after one decade, one‐quarter were disturbed after two decades, and 11% were still disturbed after three decades after initial disturbance (Jorgenson 2018).

Moist and dry vegetation types showed the most long‐term damage. In contrast, wet vegetation types recovered relatively quickly because shrubs were uncommon in these types, and the sedges were protected from disturbance by being frozen solid in the saturated soils. Riparian shrublands and *Dryas* river terraces recovered well even after severe initial disturbance because the well‐drained river gravel substrate did not subside, and because deciduous shrubs are adapted to disturbance regimes and re‐grew well (Felix et al. 1992).

**Fig. 7:**
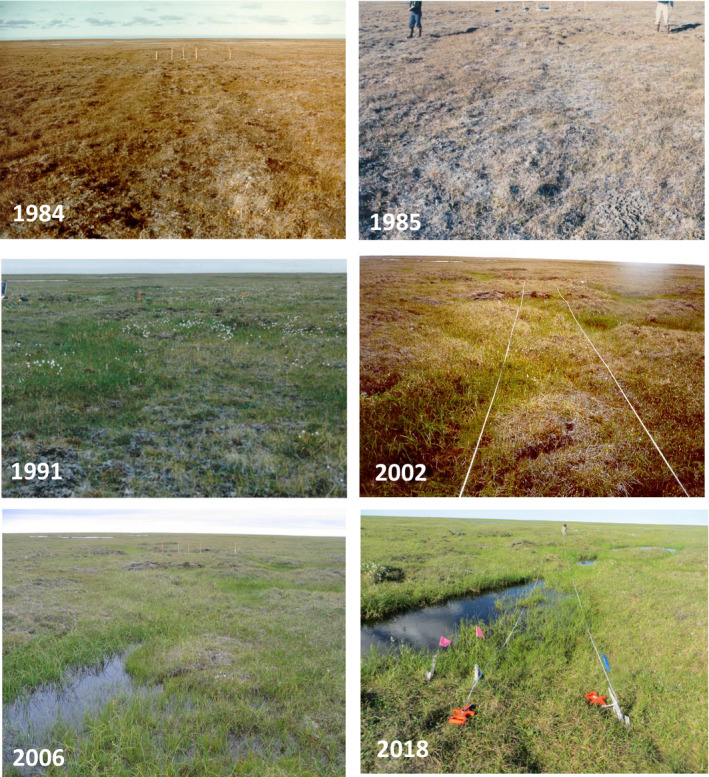
Repeat photographs of a study plot affected by thermokarst on a camp‐move trail on sedge–*Dryas* tundra in the 1002 Area of the Arctic National Wildlife Refuge (updated from Jorgenson et al. 2010). Parallel ruts and crushed vegetation were evident in 1984, the summer following disturbance (top left). By 2002, a network of sedge‐filled troughs had developed where thawing ice wedges caused ground subsidence. The thermokarst pits continued to expand and deepen through 2018 (photos: U.S. Fish & Wildlife Service).

Moist sedge–*Dryas* tundra (10% of 1002 Area, Fig. 7, 1984) recovered the least of any vegetation type after 34 yr (Fig. 7, 2018). This vegetation type occurs on ice‐rich permafrost, and has abundant frost boils with considerable micro‐relief disrupting the insulating blanket of organic soil, vegetation, and snow cover. Medium and highly disturbed moist sedge–willow tundra (37% of 1002 Area, Table 3) also occurs mainly on ice‐rich permafrost, and had longer lasting disturbance than tussock tundra (29% of 1002 Area; Jorgenson et al. 2010).

Plant species were differentially sensitive to vehicle disturbance. Species with the poorest recovery were evergreen shrubs (including Labrador tea [*Rhododendron tomentosum* ssp. *decumbens*], low‐bush cranberry [*Vaccinium vitis‐idaea*], and mountain avens [*Dryas integrifolia*]), deciduous shrubs (including dwarf birch [*Betula nana*] and dwarf willows [e.g., *Salix phlebophylla*, *S. reticulata*, *S. arctica*]), cotton‐grass tussocks (*Eriophorum vaginatum*), some mosses (particularly *Sphagnum* spp. and feather mosses such as *Tomentypnum nitens*), and all lichens (Jorgenson et al. 2010). Some vascular plant and moss species appeared to be particularly sensitive to compression of the “depth hoar” snow layer at the base of the snowpack (Walker et al. 1987).

### 3D‐seismic impacts to vegetation

Claims have been made that current 3D‐seismic methods cause insignificant impacts to the tundra compared to the 2D surveys of the 1980s (e.g., Ruskin 27 July 2018). However, a BLM Environmental Assessment stated that “seismic exploration may vary from having no observable effects in some situations to damaging vegetation to the extent that it may take years or even decades to heal. These impacts occur despite existing stipulations on operations, and cannot be further mitigated, given the types of equipment currently used.” (Bureau of Land Management 2008).

**Fig. 8:**
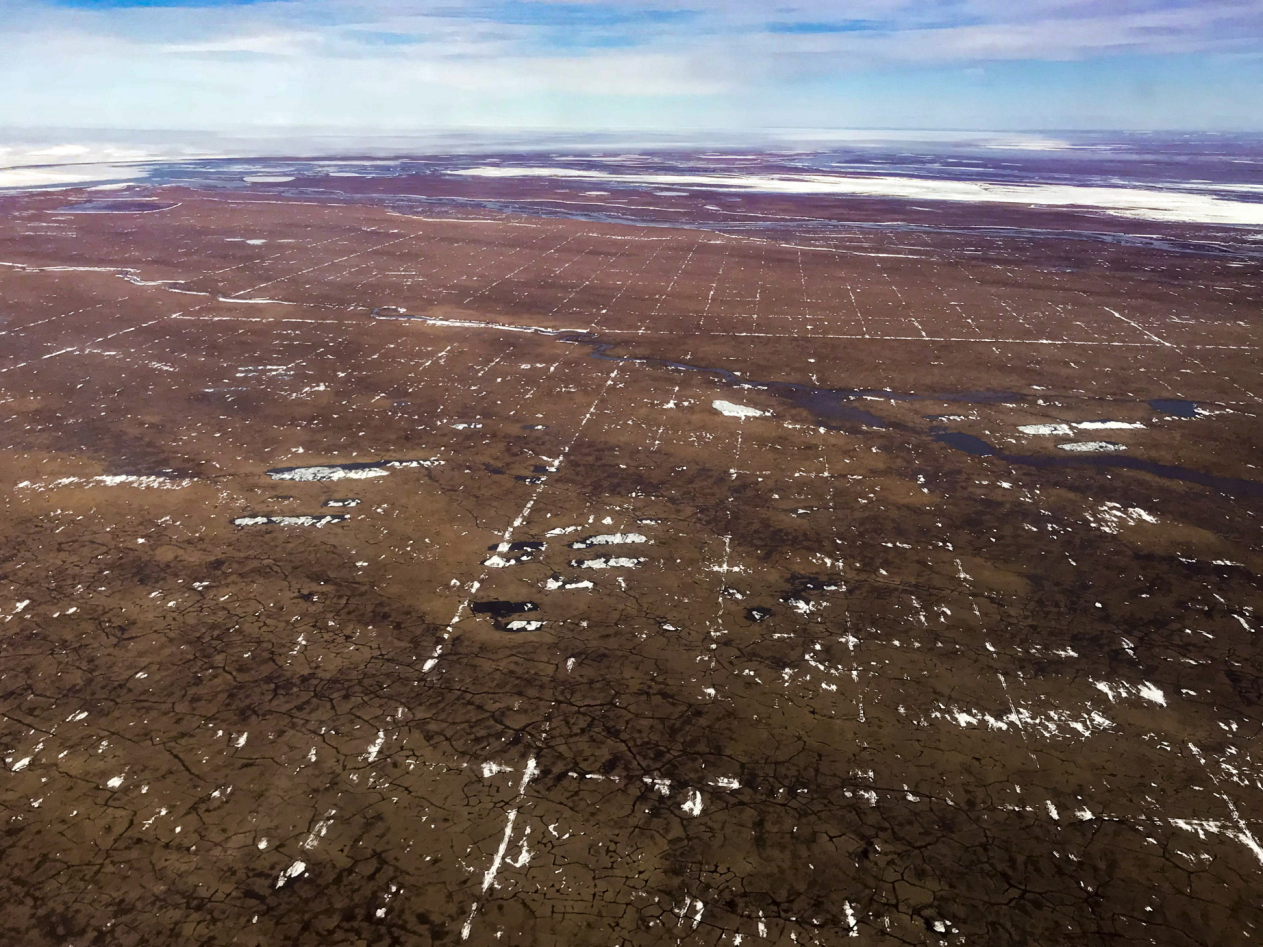
Early spring view of tracks left by a 3D‐seismic survey conducted in winter of 2017–2018 on State of Alaska lands along the western boundary of the Arctic National Wildlife Refuge, near the delta of the Canning River. The spacing of the trails was 200 × 400 m, one‐half of the 200 × 200 m proposed by SAExploration (2018) (photo: M. Nolan).

While there have been some improvements in technology, there is considerable evidence that 3D‐seismic surveys leave damaged and compressed trails similar to those of the 1980s (Appendix S1: Fig. S16, S17) and that these impacts accumulate on landscapes over time. Fig. 8 demonstrates the visibility of first‐year trails near the Canning River adjacent to the 1002 Area. The much larger area impacted by proposed 3D trails, larger sizes of vehicles, and warmer permafrost temperatures indicate that the total impacts of the proposed 3D‐seismic surveys in the 1002 Area would likely be much greater than those created by the 1984–1985 2D surveys.

We estimated the length and area of trails on each vegetation type under the proposed 3D exploration of the 1002 area by using the proportion of that type covered by the 1984–1985 seismic exploration in the same area (Table 2). The total estimated area with initial low‐level disturbance (511 km^2^) vs. medium‐to‐high level disturbance (122 km^2^) is based on the proportions of those disturbance levels found in each vegetation type in the initial studies of the 1984–1985 2D‐seismic exploration (Raynolds and Felix 1989). Over 6,000 km of trail would be expected to still be visible 10 yr after disturbance. More precise estimates would have to account for any difference in vehicles, regulations, snow depths, and permafrost temperatures.

There are few studies documenting impacts of 3D‐seismic exploration on tundra, but all show results similar to the 2D studies in the Arctic NWR. A study of impacts to upland tundra from seismic exploration on the Mackenzie River Delta, Canada, reported that initial impacts were similar to or somewhat greater than those reported from 2D surveys in the same area 30 yr previously (Kemper and Macdonald 2009*a*, *b*). One study of a 2001 3D‐seismic exploration program on Alaska’s central Arctic coastal plain found that 6% of seismic lines and 29% of camp‐move trails initially had medium or high‐level disturbance (Jorgenson et al. 2003). No long‐term follow‐up studies were done. A study of disturbance from 1998 3D‐seismic exploration in the flat coastal plain of the NPR‐A found that 4% of seismic lines were still disturbed after six years and 2% after 15 yr. In addition, 63% of the camp‐move trails were still disturbed after six years and 20% after 15 years (Yokel and Ver Hoef 2014). A study of repeated 2D exploration in the flat Colville River delta in 1992, 1993, and 1995 and from 3D work in 1996 found high levels of disturbance persisted on 1% of the sites surveyed (Jorgenson and Roth 1996). The same study found a much higher density of trails associated with the 3D operations and difficulty in quantifying the number of random stray trails that were not part of the seismic lines or camp‐move trails. Some areas were surveyed several times by different companies, resulting in a maze of seismic trails, camp trails, and ice roads that were difficult to identify by type and year of origin.

The results of these studies were similar to the results of studies of 2D‐seismic trails in terms of the percentage of trails with persistent disturbance, and the relatively greater impacts from camp‐move trails than seismic vehicle trails. The main difference was the greater density of the 3D trails. Repeat 3D surveys of the same areas are common, partially related to 4D analyses that examine time‐series of changes to known hydrocarbon deposits. Some repetition was also caused by the proprietary nature of the surveys, encouraging different companies to gather data and conduct analyses independently. Even trail locations are considered proprietary information, so no database exists to allow researchers to identify patches of tundra that have been driven on or not. In practice, especially in the older parts of the Prudhoe Bay oilfield, the result of these repeated, dense, seismic surveys is that little tundra is left undisturbed. Although the disturbance is relatively minor in most areas, the cumulative effects of the minor disturbance can result in long‐term changes to species composition of vegetation communities over very large areas (Raynolds et al. 2014*b*).

### Active layer and hydrology

Vehicle traffic strongly influenced the active layer and hydrology of permafrost landscapes through its effects on microtopography, as the land surface and ecosystems continually adjust to microscale thermal and hydrological changes (Liljedahl et al. 2016). In the 1002 Area, mechanical disturbance by seismic vehicles often broke tussocks, displaced loose soil, and disturbed the integrity of the vegetative mat, allowing solar radiation to heat the mineral soil during the following summer, deepening the active layer and thawing permafrost (Felix et al. 1992). The soil active layer was deeper on about 50% of the disturbed plots than on adjacent control areas after 10 yr (1984–1994), indicating that deeper thaw and ecosystem changes were still ongoing (Jorgenson et al. 2010). Resulting thaw settlement led to changes in surface hydrology and caused recovery patterns to shift away from the original site conditions toward new, wetter plant communities, making some trails visible for decades (Fig. 7, Appendix S1: Figs. S14, S15; Jorgenson et al. 2010).

**Fig. 9:**
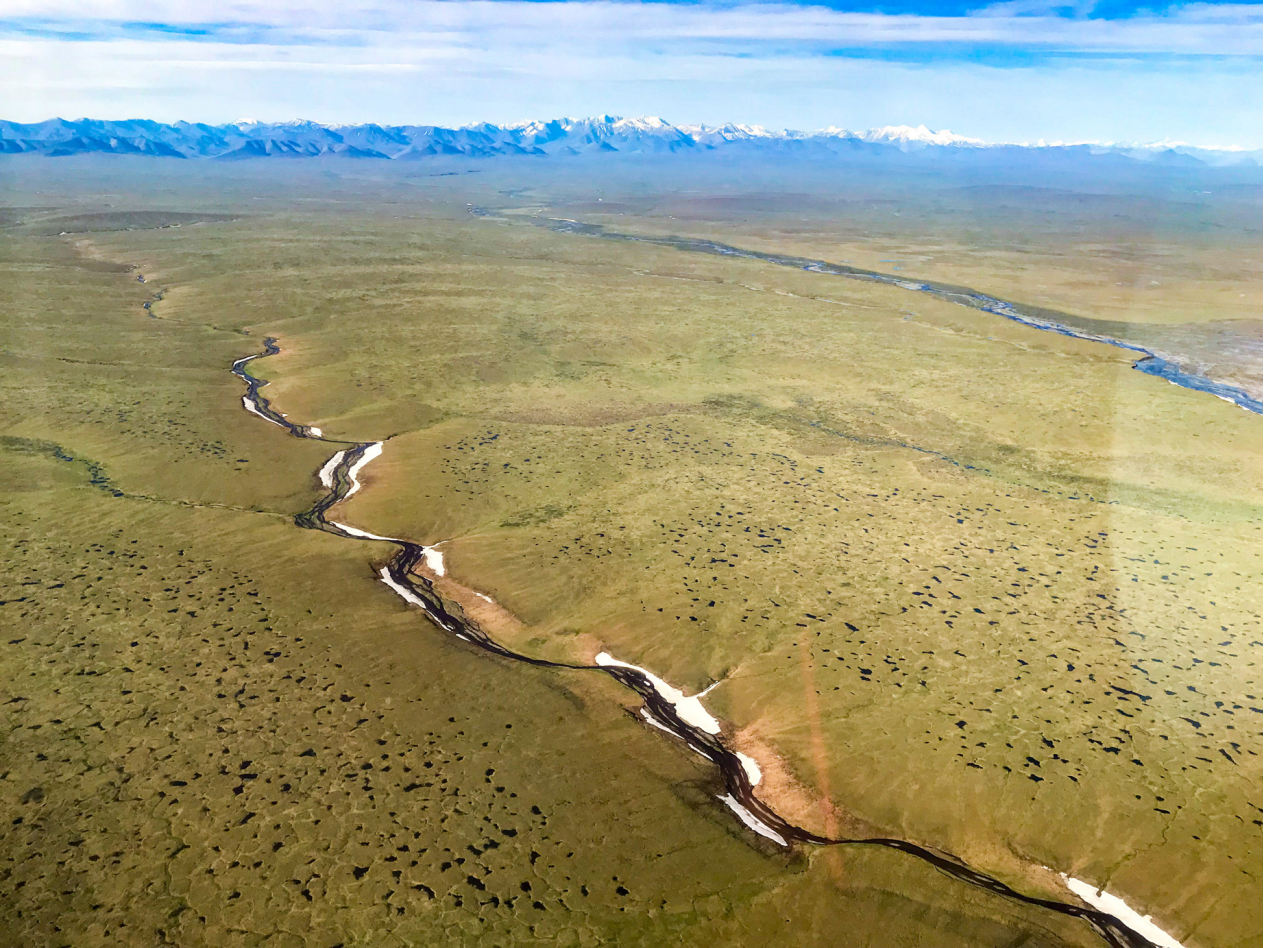
Thermokarst pits in undisturbed terrain of the 1002 Area of the Arctic National Wildlife Refuge, east of the Jago River. The numerous small thermokarst ponds (up to ~5 m diameter) are caused by thawing of the upper surface of ice wedges that separate the ice‐wedge polygons. Thermokarst such as this has recently become widespread across large areas of undisturbed tundra in northern Alaska, and is now common on upland surfaces of the 1002 Area (Jorgenson et al. 2018) (photo: M. Nolan).

Lingering snow and water in seismic trails in springtime promoted ponding of water on the tundra surface (Fig. 8, Appendix S1: Figs. S16, S17), and channeling of water along the tracks. This altered the micro‐surface energy balance, which affected the underlying active‐layer and permafrost. In some sensitive landscapes, this can trigger melting of ice in the permafrost (Jones et al. 2013), leading to thermokarst and thermal erosion of the trails. Thermokarst troughs and pits in flat areas fragment landscape‐scale water flow and storage (Liljedahl et al. 2016). Climate warming has caused similar thermokarst across the Arctic tundra region in the last decades, even in areas without traffic impacts (Fig. 9), resulting in a more variable snow cover (Liljedahl et al. 2016), and therefore higher risk of trail disturbance.

Thermokarst occurred due to trails on ice‐rich permafrost in the 1002 Area, which thawed and caused ground subsidence and formation of thermokarst depressions (most commonly, pits and troughs above degrading ice wedges) after medium‐ or high‐level initial disturbance (Appendix S1: Fig. S15, Jorgenson et al. 2018). This process was not evident in the first few years (Fig. 7, 1985), but was obvious after 7 yr (Fig. 7, 1991, 2001), and after 22 yr resulted in numerous thaw ponds and changes to topography that are likely permanent (Fig. 7, 2006, 2018). This impact is especially likely to occur on disturbed moist sedge–*Dryas* tundra, which covers 10% of the 1002 Area and occurs on ice‐rich permafrost.

Flowing water is of particular concern as it causes more erosion of permafrost and soils than stagnant water. Trails down slopes that cause sufficient disturbance to channelize surface flow can rapidly form deep gullies as a result of thermal erosion along ice wedges. This type of disturbance can rapidly expand beyond the initial trail footprint. For example, a new drainage system developed in ice‐wedge‐polygon tundra with a gentle 0.6° slope, at rates of up to 5 m/d, creating a 750‐m‐long and 4‐m‐deep gully system in four years at a site in Canada with a mean annual temperature of −15°C (Fortier et al. 2007). Increased precipitation, in conjunction with warming air and soil temperatures, has also destabilized ice‐rich permafrost terrain, resulting in mass‐wasting events through retrogressive thaw slumps (Kokelj et al. 2015). Given the hilly terrain of the 1002 Area, we expect this process to be more common than in the flatter areas to the west.

These impacts are not restricted to the trail footprints. Thermokarst depressions can interconnect, forming new surface drainage networks that can dry out perched wetlands. Thus, increased hydrologic connectivity due to expanding drainage networks can produce impacts to the landscape beyond the initial disturbance area, even years to decades after the initial disturbance. Increased sediment load from thawing permafrost would also affect downstream aquatic habitats for fish and other species.

### Permafrost

The presence of *permafrost* greatly increases the complexity of ecological responses to disturbance in the Arctic (see Appendix S1: Table S5 for glossary of italicized permafrost terms). Permafrost is continuous under land surfaces in northern Alaska, extends from 200 to 600 m in depth, and contains large amounts of ground ice, especially in its upper horizons (Kanevskiy et al. 2013). Protection of the underlying permafrost is, thus, a key consideration for any activity that could result in deepening of the active layer by altering the snow, vegetation, or surficial peat.

A major concern is that rapidly increasing permafrost temperatures due to climate change may make these tundra ecosystems more sensitive to disturbance. North Slope permafrost borehole temperatures at 20‐m depth have increased steadily since about 1990 and show some of the strongest increases anywhere in the Arctic (Romanovsky et al. 2016). Data from boreholes in the 1002 Area and Kaktovik show warming of up to 3°C since 1985 (Osterkamp and Jorgenson 2006). Recent research in the region has identified four other aspects of permafrost that are of concern for long‐term stability after disturbance from seismic exploration, including (1) the presence of an ice‐rich layer in the upper permafrost (Appendix S1: Fig. S18a), (2) widespread distribution *of ice wedges* (Appendix S1: Fig. S18b) and *ice‐wedge polygons* (Appendix S1: Figs. S19, S20), (3) the occurrence of extremely ice‐rich Pleistocene permafrost (Appendix S1: Fig. S18c), and (4) feedbacks from altered hydrology caused by permafrost degradation (e.g., Appendix S1: Fig. S15).

During early 2D‐seismic activities on the North Slope in the 1960s, tundra vegetation and soil were bulldozed to the permafrost table to create temporarily hard surfaces for trucks to drive on. This exposed the tops of ice wedges to rapid melting and extensive thermokarst formation and resulted in permanent trails, visible as a linear series of ponds or shrubby gullies on the tundra (Appendix S1: Fig. S21). Since the 1970s, seismic exploration has been confined to winter, but some disturbance still occurs (Appendix S1: Figs S11, S12, S14, S15). Much of the less severe but persistent disturbance on seismic trails made in 1984–1985 in the 1002 Area can be attributed to the thawing of *segregated ice* in the upper permafrost and to ice‐wedge degradation (e.g., Fig. 7).

The upper layer of permafrost just below the seasonally thawed *active layer* tends to be ice rich from the accumulation of segregated ice in fine‐grained soils. The thickness of the active layer in the study area commonly varied from 0.2 to 0.3 m in peat, to more than 1 m in sandy areas with little vegetation (Kanevskiy et al. 2013). Vegetation growth and peat accumulation above mineral soils lead to decreased active‐layer thickness and formation of the ice‐rich *intermediate layer* of the upper permafrost (Shur 1988). *Ice‐rich permafrost* has large *thaw settlement* potential (Pullman et al. 2007, Shur and Jorgenson 2007, Kanevskiy et al. 2013, Jorgenson et al. 2015*b*). In fine‐grained surficial deposits, the ice‐rich zone can be 60–80% segregated ice (by volume; e.g., Appendix S1: Fig. S18a). In samples from the 1002 Area, the top 30 cm of soil contained up to 25% ice in tussock tundra and up to 50% ice in moist‐sedge tundra (Felix and Raynolds 1989*b*). After disturbance, thawing of ice‐rich permafrost leads to newly thawed soil being incorporated into the thickening active layer, and the active layer equilibrates to the new surface conditions. Moderate surface disturbance can lead to an increase in thaw depths to 80 cm, with typical thaw settlement potential of 10–40 cm depending on the terrain type (Pullman et al. 2007).

Thawing of ice‐rich upper permafrost was frequently observed at medium to high levels of disturbance following the 1984–1985 seismic surveys in the 1002 Area. Thaw depths were typically 10–15 cm deeper in tracks than in adjacent reference areas during the first decade (Jorgenson et al. 2010). Plots with greater amounts of ice in the upper permafrost tended to have greater soil subsidence and higher levels of disturbance (Fig. 7; Jorgenson et al. 2010)*.*

Ice wedges (Appendix S1: Fig. S18b), a common and widespread type of *massive ice*, in northern Alaska are typically 2–3 m across the top and extend 2–4 m downward (Kanevskiy et al. 2017). They occur in a polygonal network, forming a matrix of massive ice framing ice‐wedge polygons (Appendix S1: Figs. S19, S20). The size and volume of ice wedges vary greatly by terrain type and age, typically occupying 10–20% of the volume of the top 3 m of permafrost (Kanevskiy et al. 2013).

Because ice wedges form just below the active layer, they are particularly sensitive to disturbance and climate warming. Degradation of ice wedges can lead to water‐filled *ice‐wedge‐troughs* and *thermokarst pits* (Fig. 9) in flat terrain. This is usually triggered by an increase in the active‐layer thickness, which can occur during exceptionally warm and/or wet summers or as a result of flooding or other disturbance. In the absence of significant lateral flows, the process usually stabilizes as thermokarst pits and water‐filled ice‐wedge troughs are colonized by rapidly growing aquatic algae, sedges, and mosses, creating organic layers that protect the ice‐wedge from further thaw (Jorgenson et al. 2006, Jorgenson et al. 2015*b*). During stabilization, a new *intermediate layer* of ice‐rich soil develops at the base of the active layer, and the ice‐wedges resume growth, indicating a somewhat cyclic and reversible process, although the land surface does not return to its pre‐thaw condition and the deepest thermokarst depressions may persist for centuries (Kanevskiy et al. 2017).

In recent years, ice‐wedge thermokarst has become much more widespread in tundra landscapes across the circumpolar Arctic, corresponding to increases in permafrost temperatures and deeper summer thaw (Osterkamp and Jorgenson 2006, Jorgenson et al. 2015*b*, Liljedahl et al. 2016). Thermokarst also occurs in association with tundra wildfires (Jones et al. 2015) and human activities (Raynolds et al. 2014*a*). Ice‐wedge degradation has dramatically increased since 1990 in the central and eastern parts of the North Slope (Jorgenson et al. 2006, 2015*a*, Raynolds et al. 2014*a*, Frost et al. 2018). The extent of thermokarst across the 1002 Area increased since 1984–1985 (e.g., Fig. 9), presumably due to the warming climate and positive feedbacks from impounded surface water. A recent remote‐sensing interpretation of landscape change (1949–2007) estimated that ice‐wedge degradation had changed 12% of the Arctic tundra within the northern foothills and coastal plain of the Arctic NWR (Jorgenson et al. 2018).

The most vulnerable type of permafrost is extremely ice‐ and organic‐rich silt deposits of Pleistocene age, called *yedoma* (Pullman et al. 2007). Yedoma likely occurs in the western portion of the 1002 Area (Appendix S1: Fig. S22) (Kanevskiy et al. 2011), but its distribution and characteristics in the 1002 Area are poorly known. For example, the area shown in Fig. 9 is thought to be underlain by yedoma. These Pleistocene‐age deposits elsewhere on the North Slope can be more than 40 m thick and contain large syngenetic ice wedges that span the whole yedoma sequence, with potential thaw settlement of 10–20 m or more if the deposits were to thaw completely (Appendix S1: Fig. S18) (Kanevskiy et al. 2011). While disturbance from winter seismic exploration is unlikely to lead to complete degradation of the ice in yedoma deposits, there is potential for severe disturbance to cause *active‐layer failure*, resulting in landslides, thaw slumps, or deep thermal erosion gullies on slopes. Examples of these features include the numerous thaw slumps and thermal erosion gullies in yedoma along Camden Bay associated with coastal erosion (Jones et al. 2009), and landslides that occurred after fire in the Anaktuvuk River area (Jones et al. 2015). To date, the only example of oil exploration on yedoma was the exploratory drilling during the 1940s to 1950s in the Naval Petroleum Reserve Number 4 (now the NPR‐A), where very severe subsidence was noted at several wells during cleanup operations in the 1980s (e.g., Lawson 1982).

## Data Gaps

Our review demonstrates a number of questions that cannot be answered with existing studies. (1) How much snow is needed to minimize the impacts of 3D‐seismic vehicles in different terrain and vegetation types, and with vehicles of different ground pressures? (2) What are the initial and longer‐term impacts of 3D‐seismic exploration under current ADNR permitting and under BLM permitting? (3) What are the cumulative impacts of 3D‐seismic work, in light of what we know about climate change and the types of industrial development that likely follow exploration? (4) What are the spatial and temporal distributions of snow in the 1002 Area, and how do they relate to regulatory minimums? (5) What parts of the 1002 Area have the most vulnerable vegetation and permafrost types that should be considered in managing winter traffic to minimize impacts? We identified the following data gaps that should be addressed in order to answer these questions.

### Studies of 3D seismic impacts

There is a critical need for information specific to modern 3D‐seismic methods for managers of the 1002 Area and other areas of the Arctic where these surveys are being proposed and conducted. Most of the studies reported here pertain to 2D‐seismic techniques in the 1002 Area, with much less information available regarding damage and recovery from 3D‐seismic surveys (Bureau of Land Management 2008), and even less on the impacts of 3D surveys in hilly terrain. Several broad topics include (1) the relative impacts of different vehicles in current use on different terrains, vegetation types, and snow conditions; (2) the effectiveness of different approaches to regulation: specific opening and closing dates (ADNR) vs. self‐monitoring (BLM); (3) comparing the impacts from past seismic trails to current ones in light of the rapidly warming Arctic climate and permafrost; (4) the hydrological effects of ice‐wedge degradation in hilly landscapes; and (5) the long‐term effects of low‐level but very extensive impacts to tundra ecosystems.

Many of these suggested studies could be done using trails already created by recent 3D‐seismic programs in the Prudhoe Bay area and foothills to the south. However, concurrent impact data collection should be required as part of proposed future seismic exploration. Data collected by monitors who measured snow depth and observed vehicle impacts at the time of occurrence were critical in the analysis of impacts following the 1984–1985 2D surveys in the 1002 Area. Currently, fly‐by inspections by land management agencies are done soon after exploration to look for fuel spills, garbage, and trail damage, but little on‐the‐ground‐monitoring of snow and terrain conditions is done to determine conditions before or at the time of the surveys, or to determine long‐term terrain and vegetation damage and recovery. Real‐time ground‐based monitoring during current and proposed 3D‐seismic surveys combined with very‐high‐resolution imagery would provide valuable information for continuing management of these activities.

### Climate, snow cover and ground temperature measurements

Continuous, accurate records of air temperature, soil temperature, precipitation, and snow depth across the 1002 Area are needed to provide data on which to base regulations. This would require a network of climate sampling sites located in characteristic areas. In addition, data on the spatial and temporal variability of snow depth and density should be collected for several years to statistically determine averages and patterns, using a combination of aerial imagery (Nolan et al. 2015), radar remote sensing (Wendleder et al. 2019), ground sampling (Sturm 2018, and modeling (König and Sturm 1998, Liston and Sturm, 2002). Similarly, soil moisture and depth of freeze could be monitored remotely using InSAR measurements from satellites and aircraft (Rabus et al. 2010, Chen et al. 2019). This would provide maps for planning purposes as well as near‐real‐time data for monitors and operators on the ground. Current data are especially important as we cannot rely on sparse data from the past; the warming climate is likely to lead to previously unknown conditions.

### Maps of existing landscape characteristics

New, finer resolution maps of the 1002 Area are needed to adequately regulate seismic exploration. All currently available landcover maps are based on 30‐m Landsat imagery (Jorgenson et al. 1994), a scale that is coarser than optimal for management purposes. The landcover mapping needs to be tied to key landscape information to create a GIS database relevant to regulators, including local climates, meso‐ and micro‐scale topography, snow, hydrology, soils, and permafrost characteristics. Sub‐meter resolution mapping is now possible, using very‐high‐resolution optical satellites, airplane or drone imagery, field measurements, and remote sensing techniques that use modeling and artificial intelligence (e.g., Zhang et al. 2018).

There is very little information on the magnitude and distribution of ground ice in the 1002 Area, information that is essential for mitigating the impacts of seismic exploration. The size, abundance, and distribution of ice wedges across the varying terrain types need to be sampled. The likelihood that climate warming has increased the sensitivity of ice wedges to disturbance also warrants further study. To highlight the basis for our concern, we estimate that almost 4 million near‐surface ice wedges would be crossed by the ~63,000 km of trails resulting from the seismic work proposed by SAExploration, based on an average distance of 15 m between ice wedges. Refinement of the 1002‐Area portion of the ArcticDEM (produced at 2‐m resolution, with 0.1‐m vertical accuracy; Porter 2018), to resolve ice‐wedge polygon topography and especially troughs would be helpful. LiDAR specifically flown for the area would produce detailed elevation information, while also providing information about vegetation height.

The eastern and western parts of the 1002 Area were shown to have different levels of disturbance from 2D‐seismic exploration, due to different topography (Raynolds and Felix 1989). Just as ADNR divided the central North Slope region into four different areas for regulating winter vehicle traffic, there may be reasons to divide the 1002 Area to better manage trail impacts. Updated vegetation and permafrost maps are needed to identify practical regulatory units with distinct snow, landscape and vegetation characteristics relevant to winter vehicle traffic.

## Conclusion

Every year, large 3D‐seismic surveys are conducted across northern Alaska and elsewhere in the Arctic. Although they create the most extensive impacts related to Arctic oil and gas production, there have been no studies of their effects in the last 20 yr, and the impacts of these activities have been assumed to be minor. This review shows that this conclusion is not supported by the scientific studies currently available.

The known landscape impacts from 2D winter seismic vehicle traffic are well documented. Initial impacts include compression of snow, breakage of vegetation and compression of moss mats, and compression and abrasion of microrelief. Due to resulting subsidence and changes in hydrology, these impacts led to essentially permanent changes in a small percentage of >30‐yr‐old seismic trails and a greater percentage of the camp‐move trails in the 1002 Area of the Arctic NWR (3% and 5%, respectively; Jorgenson 2018). The proposed 3D‐seismic exploration in the same area would create over 63,000 km of seismic trails and over 580 km of camp‐move trails, expected to result in 122 km^2^ of medium to high‐level initial disturbance.

The steeper and more heterogeneous terrain in the 1002 Area increases the likelihood of impacts compared to 3D‐seismic exploration in flatter, wetter areas of the North Slope. Extensive ice‐rich permafrost and warming soil temperatures make the soils in the 1002 Area especially sensitive to disturbance. The risk of lateral expansion of surface disturbance beyond the trails is high. The heterogeneous snow environment, both in space and time, makes it highly likely that the proposed network of seismic and camp‐move trails could not, in many areas, meet the minimum snow‐depth standards required to protect tundra vegetation and permafrost. There would likely be significant, extensive, and long‐lasting direct, indirect, and cumulative impacts to the microtopography, hydrology, permafrost, vegetation, and local ecosystems.

Based on the long‐term studies of 2D‐seismic exploration, this would permanently change the vegetation of 19.6 km^2^, over twice the area permitted by the 2017 Tax Act to be covered by oilfield infrastructure. This is a minimal estimate of impacts, as many trails take decades to recover; the area summary does not include airstrips, fuel, or personnel resupply trails; impacts are likely to be greater than in the 1980s due to warmer permafrost; and it does not include the laterally extended effects due to erosion and hydrological connectivity. It also does not include any cumulative ecosystem effects due to the dense network of the trails, compounding effects of climate change, or any changes more subtle than a complete change in vegetation type. These changes would impact the quality of habitat for caribou, birds, fish, and other wildlife that use the Arctic NWR. It would impact villages that rely on subsistence resources from this area, and the quality of the wilderness experience of recreational visitors.

We conclude that the much denser and more extensive networks of trails, larger camps and greater number of camp sites, and more numerous and larger vehicles associated with 3D exploration, combined with a warmer climate, would create much more damage than the previous seismic survey in the 1002 Area. The impact could be reduced by decreasing the extent of trails and number of camps, either by selectively reducing the area to be surveyed, or by using other types of exploration or camp support technologies.

This conclusion emphasizes the importance of doing the necessary research beforehand in order to have as much information as possible for both operators and regulators, and then to follow those regulations and adapt when and where necessary to avoid and minimize impacts. Information about the timing, extent and depth of snow are critical. Finer‐resolution maps of vegetation, permafrost (especially ice‐rich permafrost) are necessary both as baselines to assess change and as management tools. An increased understanding of landscape trajectories following ice‐wedge degradation in flat vs. hilly ice‐rich terrain is needed. Studies of impacts of recent 3D‐seismic exploration are important to determine how to use newly collected site‐specific information to minimize impacts. Any studies conducted in relationship to the needs in the 1002 Area will also have wide value elsewhere outside the Refuge, wherever oil and gas exploration or other winter travel is being done in the Arctic.

## Supporting information

Appendix S1

## Data Availability

Data are available from the Arctic Data Center: https://doi.org/10.18739/A2B56D49C
