# Supplementary material for: Landscape impacts of 3D‐seismic surveys in the Arctic National Wildlife Refuge, Alaska
Source: Ecol Appl. 2020 May 26;30(7):e02143. doi: 10.1002/eap.2143 (PMC7583382; doi:10.1002/eap.2143)
Supplement: Supplementary file 1 — Appendix S1 [file EAP-30-e02143-s001.pdf]

Supporting Information. Raynolds, M.K., J.C. Jorgenson, M.T. Jorgenson, M. Kanevskiy, A.K. Liljedahl, M. Nolan, M. Sturm, and D.A. Walker. 2020. Landscape impacts of 3D-seismic surveys in the Arctic National Wildlife Refuge, Alaska. Ecological Applications.

Appendix S1

Table S1. Proposed 3D-seismic survey of the 1002 Area of the Arctic National Wildlife Refuge. The plan of operations specified the equipment list for each survey crew, with two crews operating at one time (SAExploration 2018).

Equipment list	# per crew	Model, Make or Capacity
Tucker Snow Cat	12	1644
Tucker Ice Cat	8	1644
Tucker Personnel Carrier	3	1600
GPS Base Station	3	Hagglund
Vibe Tender	2	Tucker Trailer
Mechanic Field Shop	1	Tucker Trailer
Node Charging Shack	3	Tucker Trailer
Recorder	1	Tucker Trailer
Taco	6	Trailer
Survival Trailer	2	Tucker Trailer
GSX Nodes	TBD	GSX-1
Batteries	TBD	BX10
Sensor	TBD	Arctic Base
AHV-IV Vibrators	12	Commander (PLS-364)
Sleigh Camp	1	150 person
Fuel Tanks/Fuel Stations	7	11,400 / 15,000 liters
Long Haul Fueler	4	15,0000 liters
Rolligons	1	
Case/Steiger Tractors	9	535
CAT Dozer	2	D7G
CAT Loader	1	977H

Table S2. Vegetation types of the 1002 Area of the Arctic National Wildlife Refuge, Alaska (Jorgenson et al. 2010).

Type	Description
Wet Sedge Tundra	Low-lying flats and drainages with the sedges *Carex aquatilis and Eriophorum angustifolium* and little moss or shrub cover. The poorly-drained soils are saturated throughout the summer and have a thick, fibrous organic horizon.
Moist Sedge-Willow Tundra	Low-lying flats and gentle slopes with the sedges *Eriophorum angustifolium* and *Carex aquatilis* and the willows *Salix pulchra* and *S. reticulata*. Mosses include *Tomentypnum nitens, Hylocomium splendens, Aulacomnium* spp., *Sphagnum* spp., and *Campylium stellatum*. On fine-grained, re-transported, glaciofluvial, and abandoned floodplain deposits. The soils have a moderately thick organic layer and are saturated at intermediate depths but generally free of surface water.
Moist Sedge-*Dryas* Tundra	Moderately well-drained sites dominated by the dwarf shrub *Dryas integrifolia* and the sedge *Carex bigelowii*, with the willows *Salix richardsonii, S. phlebophylla* and *S. reticulata* and mosses such as *Tomentypnum nitens, Hylocomium splendens, Distichium capillaceum*, and *Ditrichum flexicaule*. Forbs (e.g. *Lupinus arcticus*), lichens (e.g. *Cetraria* spp.), and horsetails (e.g. *Equisetum variegatum*) are common. Found on moist calcareous slopes and pebbly glacial and marine sediments. Notable for a hummocky surface topography, patches of exposed mineral soil, and extremely variable organic horizons resulting from active and stabilized frost boils.
Moist Tussock Tundra	Moderately well-drained slopes dominated by the tussock-forming sedge *Eriophorum vaginatum*, with shrubs *Salix pulchra, Betula nana, Ledum palustre* ssp. *decumbens* and *Vaccinium vitis-idaea*. Bryophytes included *Hylocomium splendens, Sphagnum* spp., *Aulacomnium turgidum, Ptilidium ciliare*, and *Tomentypnum nitens*. Occurs on deposits of loess or colluvial material on top of coarser, residual materials, or glacial drift.
Moist Shrub Tundra	Dominated by low and dwarf shrubs, with *Betula nana* or *Salix pulchra* and understory species similar to Tussock Tundra. In the study area, occurs only on raised areas with high-centered polygon surface morphology.
Moist Riparian Shrubland	Willow shrublands on river floodplains and stream banks, dominated by *Salix alaxensis, S. glauca* and *S. richardsonii*, commonly with a forb understory. Willows have an average height of 0.5 m and maximum of about 1.5 m. Occurs on both young floodplain deposits with mixed gravel and fine-grained material, and older terraces with a thin fine-grained alluvium layer over gravel.

Dry *Dryas* Terrace	Infrequently-flooded river terraces with *Dryas integrifolia* and other dwarf shrubs, forbs, horsetails, mosses, and lichens similar to Sedge-*Dryas* Tundra. Well-drained soils with a very thin organic mat over river deposits.

Table S3. Semi-quantitative disturbance rating system for initial impacts in vegetation types at different disturbance levels resulting from the 1984-1985 seismic vehicles in the 1002 Area of the Arctic National Wildlife Refuge (Raynolds and Felix 1989).

	Disturbance level			
Vegetation Type	0 (none)	1 (low)	2 (medium)	3 (high)
Aquatic graminoid marsh	No impact	Compression of standing dead emergent vegetation		
Wet sedge tundra	No impact	Compression of standing dead to ground surface. May include slight scuffing of higher microsites.	Obvious compression of mosses and standing dead. Trail appears wetter than surrounding area. Common scuffing of micro-relief.	Obvious track depression. During wet years, standing water apparent on the trail that is not present in surrounding area.
Moist sedge-willow tundra	No impact. May have a few widely scattered scuffed microsites.	Compression of standing dead. Some scuffing of higher microsites or frost boils if present. Less than 25% vegetation damage and broken shrubs	Obvious compression of mosses and standing dead. Trail appears wetter than surrounding area. Scuffing of micro-relief common, small patches of soil may be exposed. Vegetation damage and broken shrubs 25 – 50%	Obvious track depression, over 50% vegetation damage. Compression of mosses below water surface. During wet years standing water apparent in trail that is not present in adjacent area.
Moist sedge-*Dryas* tundra	No impact to slight scuffing of microrelief.	Compression of standing dead. May have up to 25% vegetation damage. Some scuffing of mound tops. 0 – 5% soil exposed,	Vegetation damage 25 – 50%. Exposed organic mineral soil 5 – 15%. Scraping of mound tops common.	Nearly all mound tops scraped. Over 50% vegetation damage. Over 15 % soil exposed.
Moist tussock tundra	No impact to slight scuffing of tussocks	Scuffing of tussocks or mound tops. Vegetation	Mound top destruction of tussocks over 30%.	Destruction of tussocks or mound tops continuous.

	and breakage of shrubs.	damage 5 – 25%. Exposed organic or mineral soil less than 3%.	Common mound top scuffing. Vegetation damage 25 -50%. Exposed organic or mineral soil 3 - 15%.	Ruts starting to form. Vegetation damage over 50%. Exposed soil over 15%.
Moist dwarf-shrub tundra	No impact to occasional breakage of shrubs.	Vegetation damage 5 – 25%. Less than 25% shrub canopy decrease. Scuffing of tussocks and hummocks if present.	25 – 50% vegetation damage and shrub canopy decrease. Mound top destruction of some tussocks and hummocks.	Over 50% vegetation damage and over 50% broken shrubs.
Riparian low shrub	No impact to slight breakage of shrubs.	Less than 50% shrubs broken, little impact to ground cover. Less than 25% decrease in total plant cover.	50 – 80% shrubs broken, sometimes to ground level. Some disturbance to ground cover. Total plant. cover decrease 25 – 50%.	Over 80% removal of shrub canopy. Substantial disturbance to ground cover, over 50% decrease in total plant cover.
Dry *Dryas* river terrace	No impact to few, widely scattered scuffed microsites.	Less than 30% vegetation killed. Less than 5% soil exposed	30 – 60% vegetation killed. Little disruption of vegetative mat. 5 – 15% soil exposed.	Over 60% vegetation killed, vegetative mat mostly disrupted. Over 15% soil exposed or over 50% increase in bare ground.

Table S4. Semi-quantitative disturbance rating system for long-term impacts to vegetation and soils from 1984-1985 seismic vehicles in the 1002 Area of the Arctic National Wildlife Refuge (Jorgenson, 2010).

	Disturbance level			
Type of Impact	0 (none)	1 (low)	2 (medium)	3 (high)
Decrease in plant cover	No observable change	0 – 25%	26 – 50%	> 50%
Decrease in shrub canopy	No observable change	0 – 25%	26 – 50%	> 50%
Increase in organic or mineral soil exposed	None observed	1 - 5%	6 – 15%	> 15%
Damage to microscale structure	No observable damage to scattered scuffing of tussocks or hummocks	Most tussocks or hummocks scuffed, some crushed	Most tussocks or hummocks crushed	Ruts, or crushed tussocks or hummocks nearly continuous
Trail subsidence or compression	No observable change	Slight compression of vegetation and peat; trail may be wetter than surrounding area	Trail wetter than surrounding area; thaw subsidence indistinct or patchy	Standing water on the trail that is not present in surrounding area, or trail a trough due to thaw subsidence
Change in plant species composition	No observable composition change	0 – 5% species composition change	6 – 25% species composition change	> 25% change in species composition, resulting in major change in vegetation type

Table S5. Glossary of permafrost terms, based on van Everdingen (2005).

**Active layer**	The top layer of ground subject to annual thawing and freezing in areas underlain by *permafrost*.
**Active layer failure**	A general term referring to several forms of slope failures or failure mechanisms commonly occurring in the active layer overlying permafrost (e.g., active-layer detachment slides).
**Continuous permafrost**	*Permafrost* occurring everywhere beneath the exposed land surface throughout a geographic region. The term generally refers to areas where more than 90 % of the ground surface is underlain by permafrost.
**Excess ice**	The volume of ice in the ground which exceeds the total pore volume that the ground would have under natural unfrozen conditions.
**Ice-rich permafrost**	*Permafros*t containing *excess ice* (Figure 18).
**Ice wedge**	A massive, generally wedge-shaped body with its apex pointing downward, composed of foliated or vertically banded, commonly white, ice. Width of ice wedges varies from several cm to many meters, and sometimes wedge ice can occupy more than 50% of volume of the upper permafrost (Figure S18b)
**Ice-wedge polygon**	A polygon outlined by ice wedges underlying its boundaries (Figures S19, S20). The size of ice-wedge polygons varies from several meters to more than 30 m across.<br>*Low-centered polygons* are low basins in the centers of the, sometimes containing shallow ponds, framed by elevated *rims* that develop above ice wedges (Figure S19). Low-centered polygons are typical of aggrading stages of ice-wedge formation<br>*High-centered polygons* have elevated centers and well-developed troughs over ice wedges, often filled with water (Figure S20). High-centered polygons usually indicate partly degraded ice wedges (Figure 9 in main paper).
**Ice-wedge trough**	The depression above an ice wedge.
**Intermediate layer**	A thin layer of ice-rich soil and organic material that forms at the base of the *active layer*, often above *ice wedges* and serves to protect the wedge from thawing. If the intermediate layer thaws, rapid melting of the top of the ice-wedge can occur leading to *thermokarst.*
**Massive ice**	Massive ice is a comprehensive term used to describe large masses of ground ice, including ice wedges, pingo ice, buried ice and large ice lenses.

**Patterned ground**	A general term for any ground surface exhibiting a discernibly ordered, more-or-less symmetrical, morphological pattern of ground and, where present, vegetation. A descriptive classification of patterned ground includes such features as nonsorted and sorted circles, nets, polygons, steps and stripes, and solifluction features. In permafrost regions, the most ubiquitous macro-form is the ice-wedge polygon (Figures S19, S20), and a common micro-form is the nonsorted circle.
**Permafrost**	Ground (soil or rock) that remains at or below 0 °C for at least two consecutive years.
**Segregated ice**	Layers or lenses of ice in permafrost that form as a result of the migration and subsequent freezing of soil pore water.
**Thaw settlement**	Compression of the ground resulting from thawing of frozen ground; in ice-rich permafrost containing excess ice, the amount of thaw settlement may be substantial.
**Thermal erosion**	The erosion of *ice-rich permafrost* by the combined thermal and mechanical action of moving water.
**Thermokarst**	The process by which characteristic landforms result from the thawing *of ice-rich permafrost* or the melting of massive ice. Landforms found in thermokarst terrain include *thermokarst pits* (Figure 9) thermokarst ponds and lakes, drained thermokarst-lake basins (alasses), and thermokarst mounds (baydzherakhs) that form as a result of ice-wedge degradation.
**Yedoma**	Ice-rich silt and organic deposits of Pleistocene age (e.g., Figure 18c).

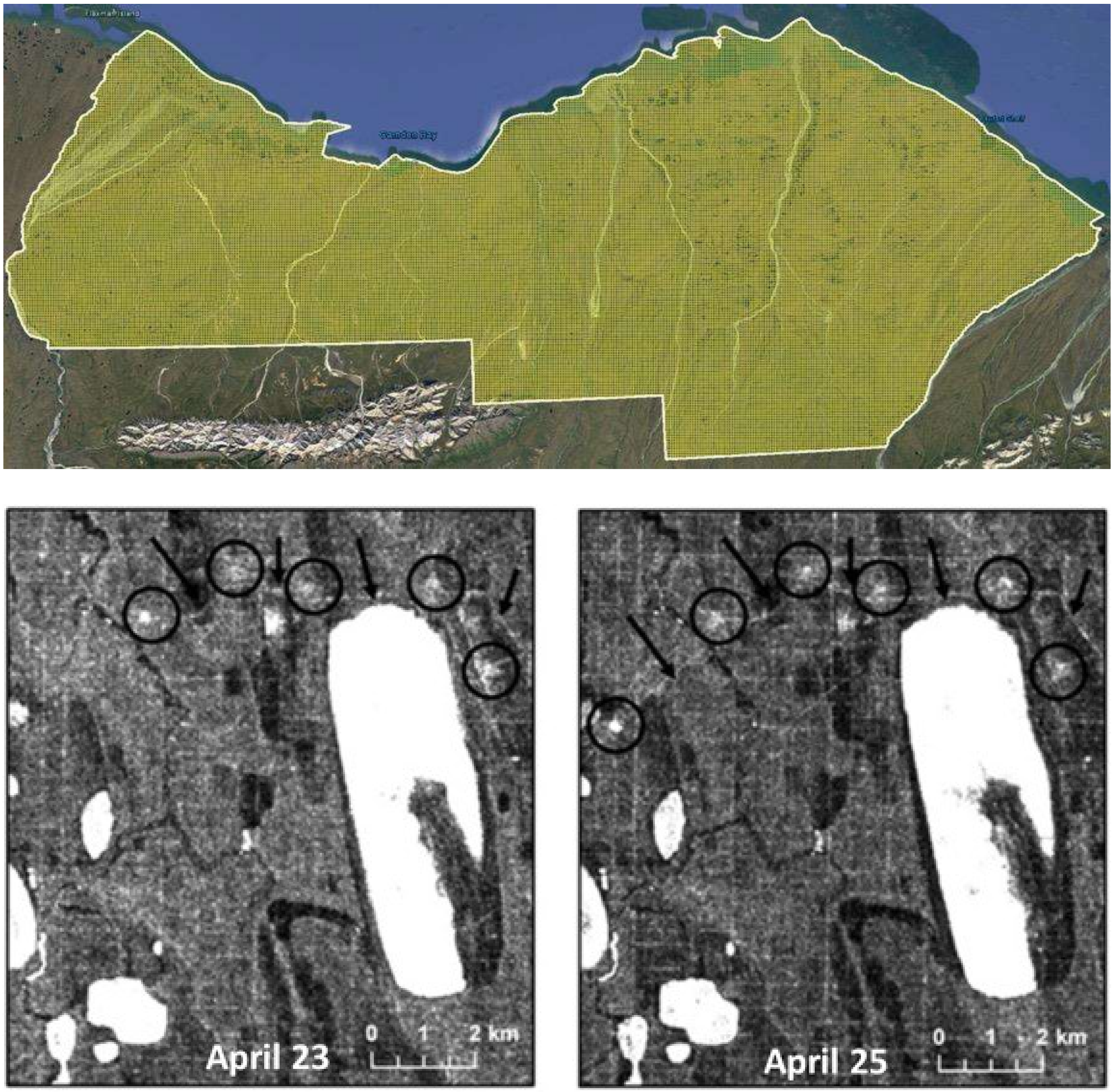

Figure S1. (Top) Grid of seismic trails at 400-m covering the 1002 Area of the Arctic National Wildlife Refuge. The proposed SAExploration survey would require twice as many trails, at 200-m intervals (Graphic: M. Nolan, Fairbanks Fodar, *http://fairbanksfodar.com/wp-content/uploads/2018/07/img_7105_acr.jpg*, Accessed 28 July, 2019).
(Bottom) Radarsat-1 SAR images from 23 April and 25 April 2006, showing a faint grid of seismic lines spaced at approximately 400 m, and a progression of camp moves (black circles) associated with a 3-D seismic survey near a large elliptical ice-covered lake south of the Lonely DEW Line Station in the NPR-A. A new campsite was added between 23 and 25 April, visible on the left side of the right photo. Note the more intense radar signal associated with the camps (circles) and camp move trails (black arrows), which corresponds to the generally more intense disturbance caused by these activities (From Jones et al. 2008).

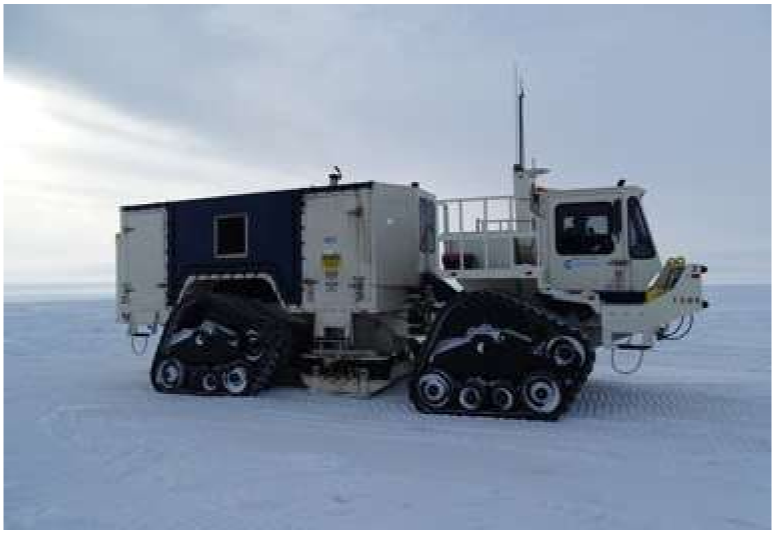

Figure S2. The rubber-tracked Inova AHV-IV Vibroseis vehicle. The vibrator pad is located between the front and rear treads of the vehicle. The SAExploration plan of operations included 12 of these per crew (SAExploration 2018). (Photo: Bureau of Land Management)

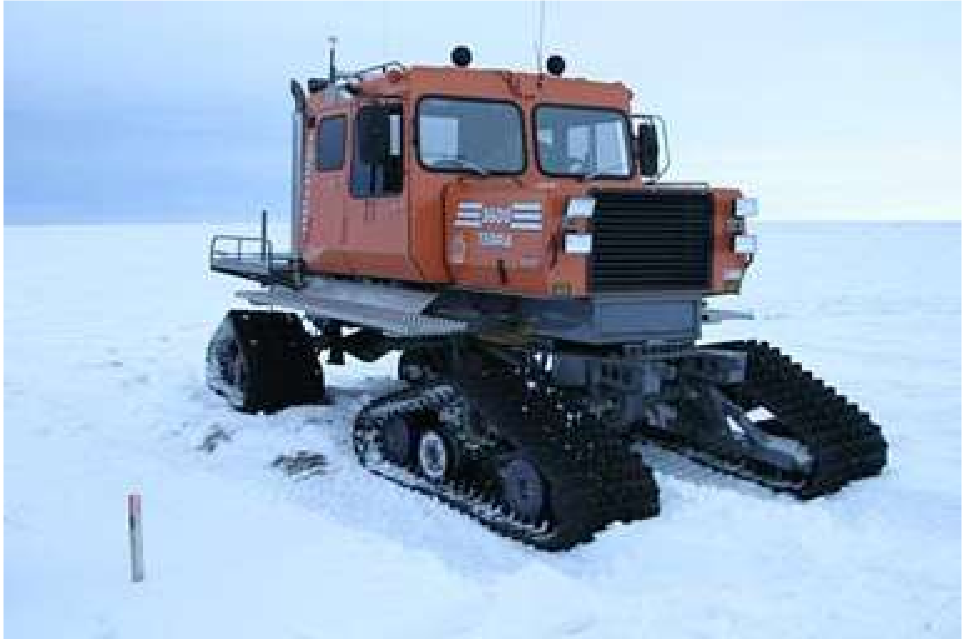

Figure S3. Tucker Sno-Cat, a cleat-tracked vehicle used to transport workers, put out and pick up geophones, prepack snow, and other uses not requiring a heavy vehicle. This is an example of seismic survey vehicles in the “Other’ category on Table 1 (main article). The SAExploration plan of operations included 12 Tucker Sno-Cats, eight Tucker Ice Cats, three Tucker personnel carriers, and a variety of Tucker trailers per crew (SAExploration 2018). (Photo: Alaska Department of Natural Resources)

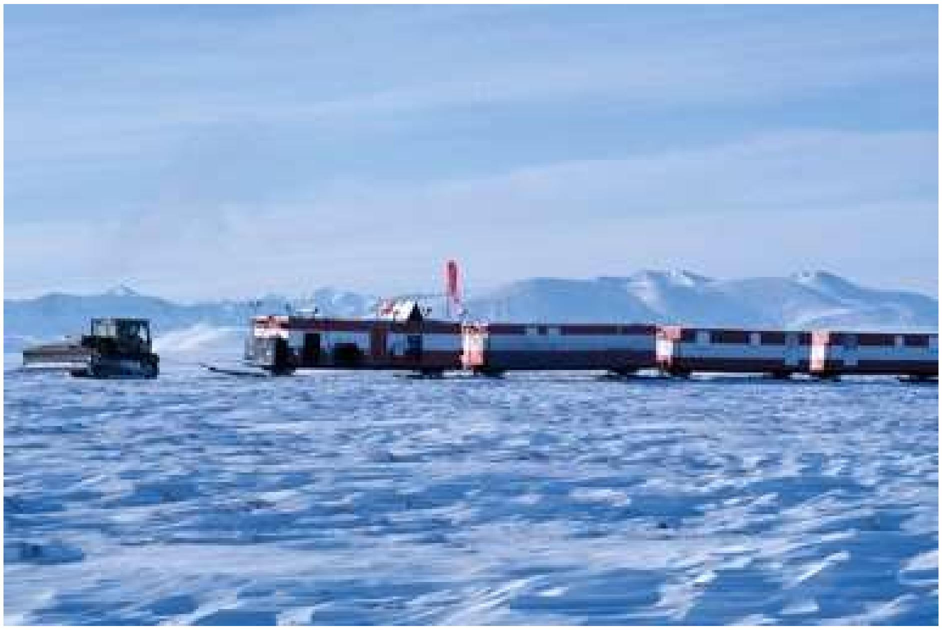

Figure S4. Cat-train of sled-mounted camp trailers pulled by a Caterpillar D7 dozer during the 1984–1985 surveys in the 1002 Area of the Arctic National Wildlife Refuge. The SAExploration plan of operations proposed one 150-person camp per crew (SAExploration 2018). The vehicles required for the mobile camps consist of 8–10 strings of 5–8 sleds pulled by large tractors. (Photo: U.S. Fish & Wildlife Service)

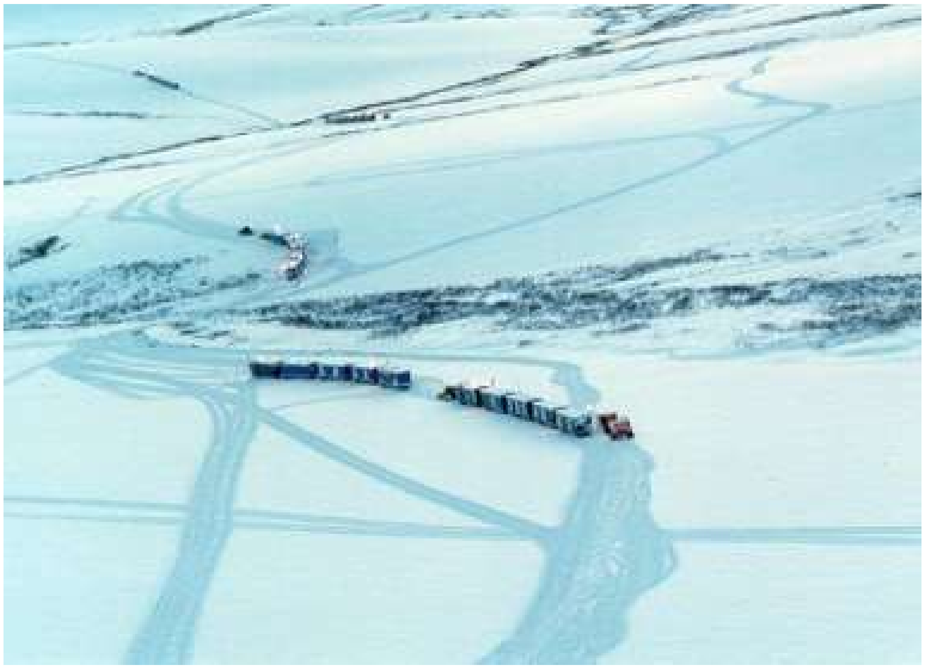

Figure S5. Five Cat-trains with sled-mounted trailers during 3D seismic exploration in hilly terrain near Kavik, west of the 1002 Area, 2001. The three Cat-trains in background apparently required two tractors per train to travel in this hilly terrain (one D7 to assist the lighter tractor), while the two trains in the foreground were waiting for tractors to return for them. (Photo: U.S. Fish & Wildlife Service)

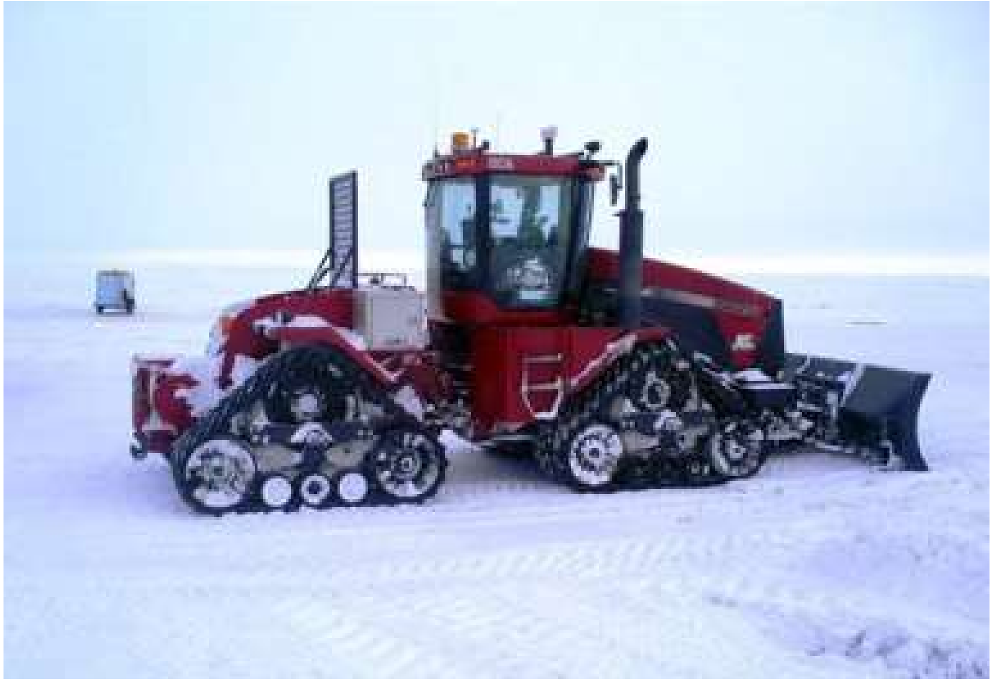

Figure S6. Steiger tractor used to haul camp trailers and other vehicles and equipment. The SAExploration plan of operations anticipated nine Case/Steiger tractors per crew (SAExploration 2018). (Photo: Alaska Department of Natural Resources)

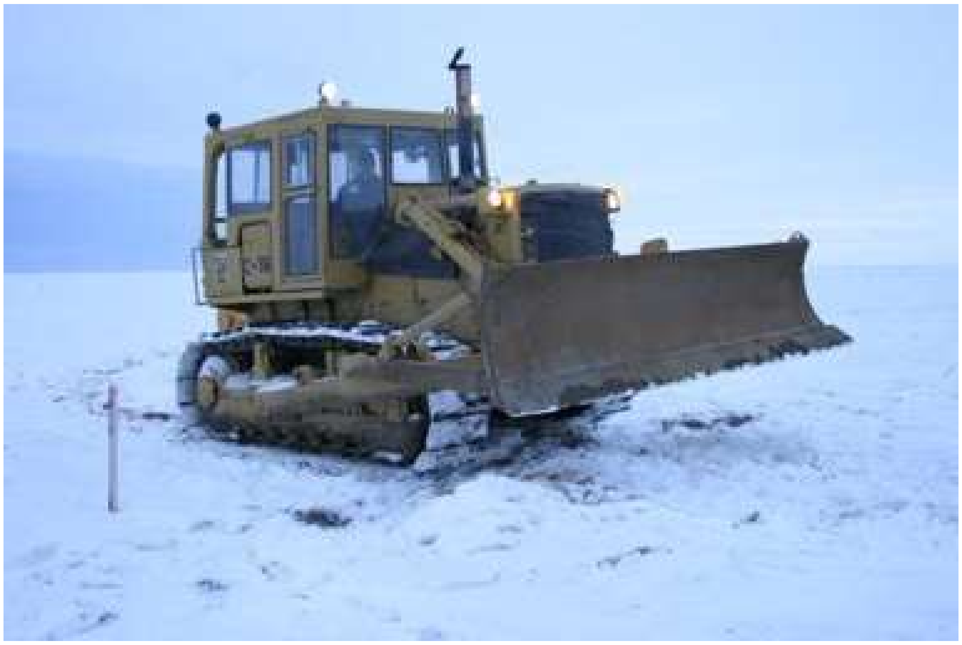

Figure S7. Caterpillar D7 steel-tracked dozer used to haul heavier Cat-train camps and equipment. The SAExploration plan of operations included two of these per crew (SAExploration 2018). However four were used per crew at GMT 3-D in 2017 in an area that was flatter than the 1002 Area. (Photo: Alaska Department of Natural Resources)

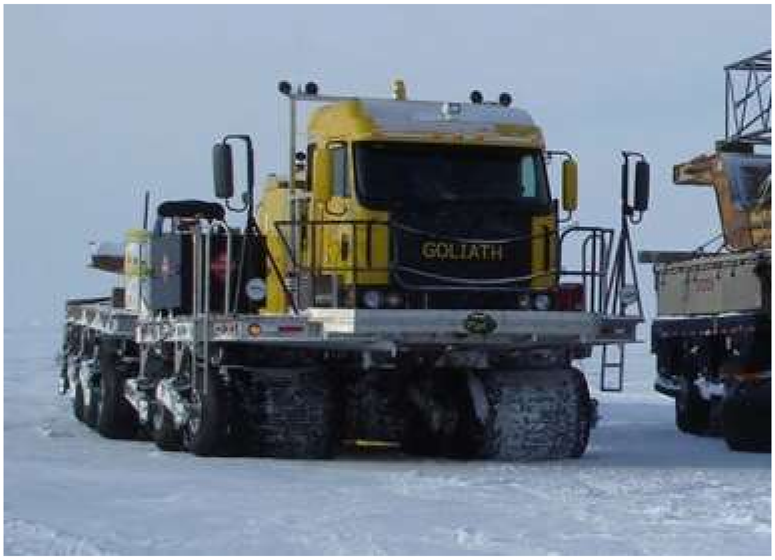

Figure S8. Rolligons - vehicles with large, low-pressure tires used by the oil industry in Canada and Alaska. The SAExploration plan of operations listed one Rolligon per crew (SAExploration 2018). (Photo: Alaska Department of Natural Resources)

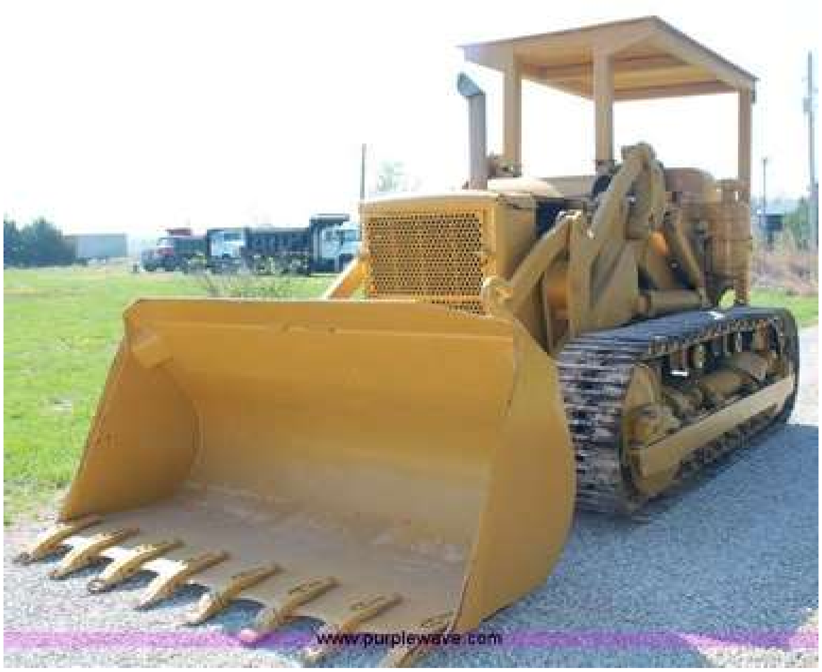

Figure S9. Caterpillar 977H tracked loader. One 977H per crew was anticipated in the SAExploration plan of operations (SAExploration 2018). (Photo: Purple Wave Auction, *https://www.purplewave.com/auction/140515/item/H6548*. Accessed 28 July 2018)

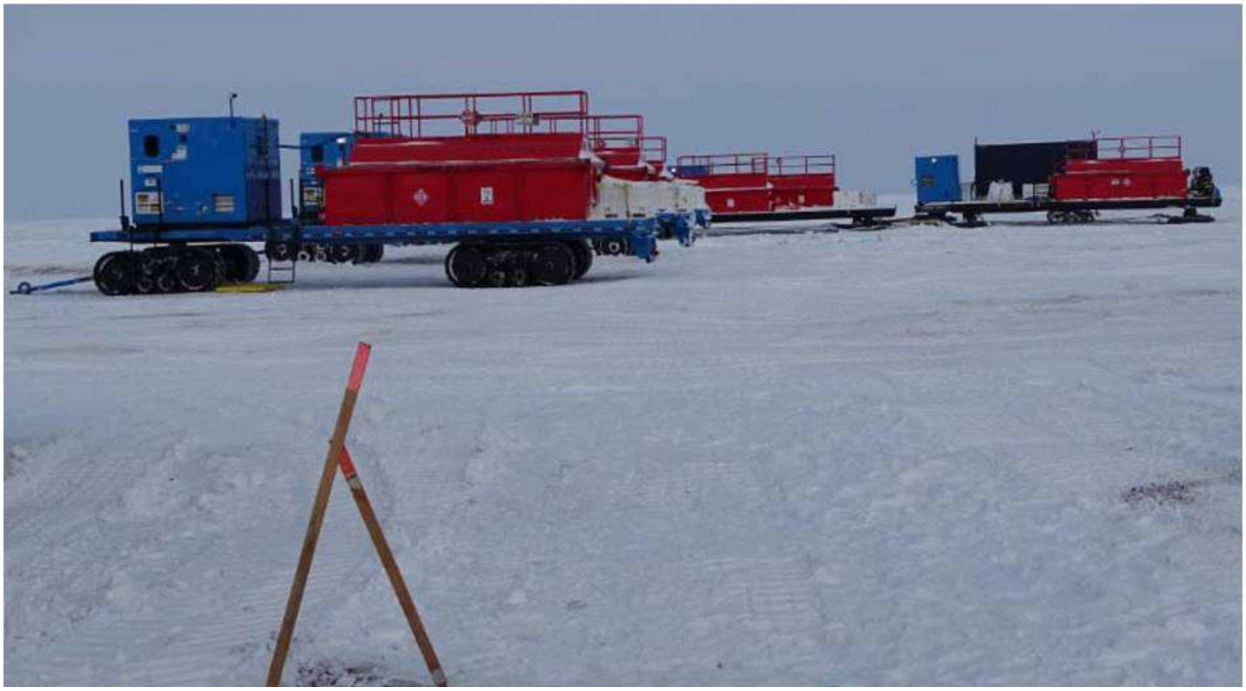

Figure S10. Fuel tanks on trailers. These trailers would be used to deliver 6,000 – 7,000 gallons of fuel every day to the camps in the 1002 Area (SAExploration 2018). (Photo: Bureau of Land Management)

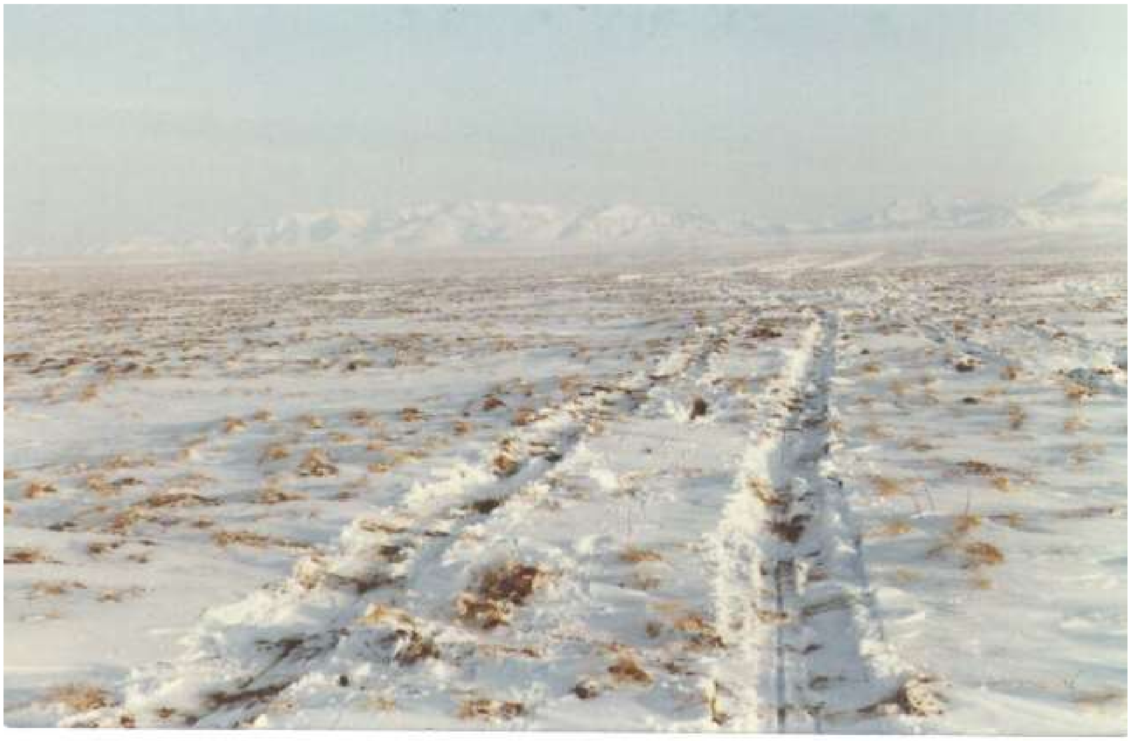

Figure S11. Camp move trail across tussock tundra vegetation in the 1002 Area of the Arctic National Wildlife Refuge, spring of 1984. (Photo: U.S. Fish & Wildlife Service)

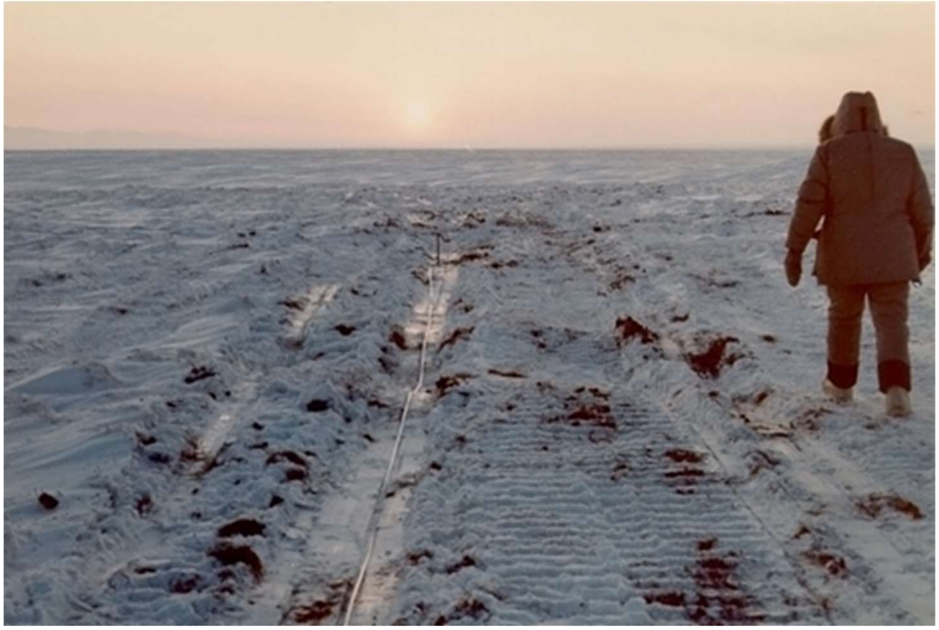

Figure S12. Trail made by Caterpillar D7 tractors and ski-mounted trailers in March 1984 in the 1002 Area of the Arctic National Wildlife Refuge. This site was still highly disturbed in 1994 (Jorgenson et al. 1996). By 2018 the trail here had subsided into a large pond due to melting of ice wedges (Jorgenson 2018). Based on the amount of exposed soil in the tracks, it is clear that there was insufficient snow to protect the tundra. (Photo: U.S. Fish & Wildlife Service)

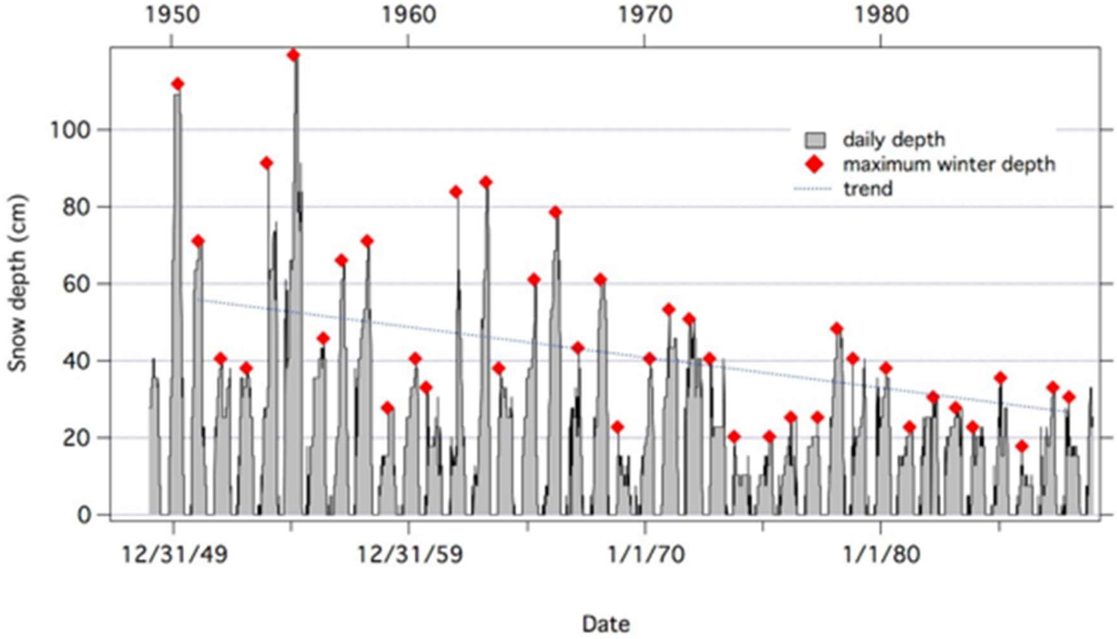

Figure S13. Snow depth records from Kaktovik Alaska, 1948-1988 (National Weather Service records). This sampling was discontinued after 1988.

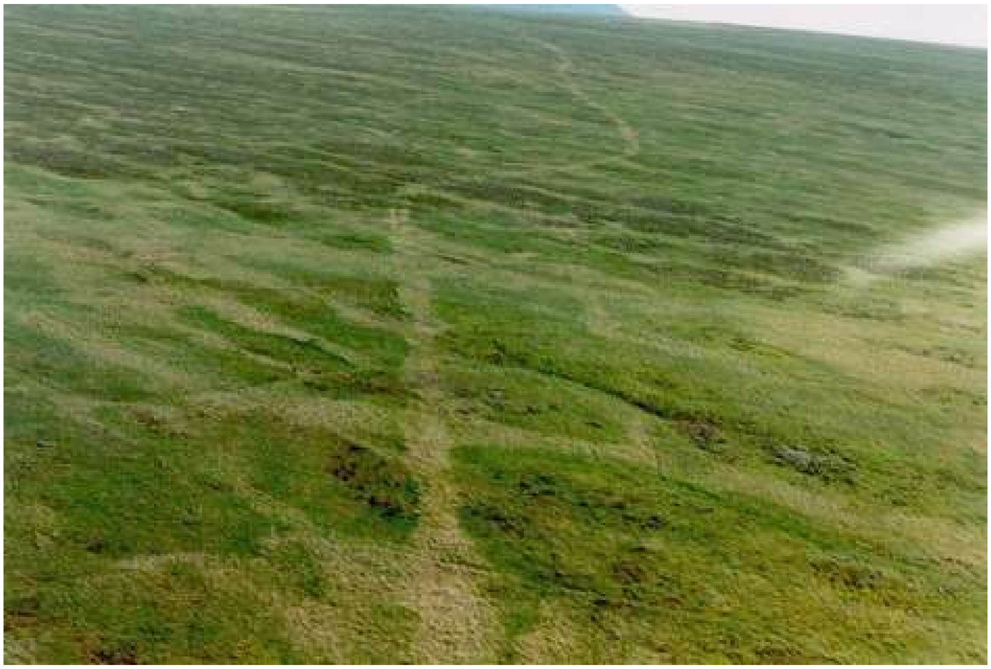

Figure S14. Camp-move trail in the 1002 Area of the Arctic National Wildlife Refuge, photographed in 1994, 10 years after it was made. This trail remained visible due to trail subsidence, a decrease in shrubs and mosses, and increase in standing dead sedge leaves (Jorgenson et al. 1996). (Photo U.S. Fish & Wildlife Service)

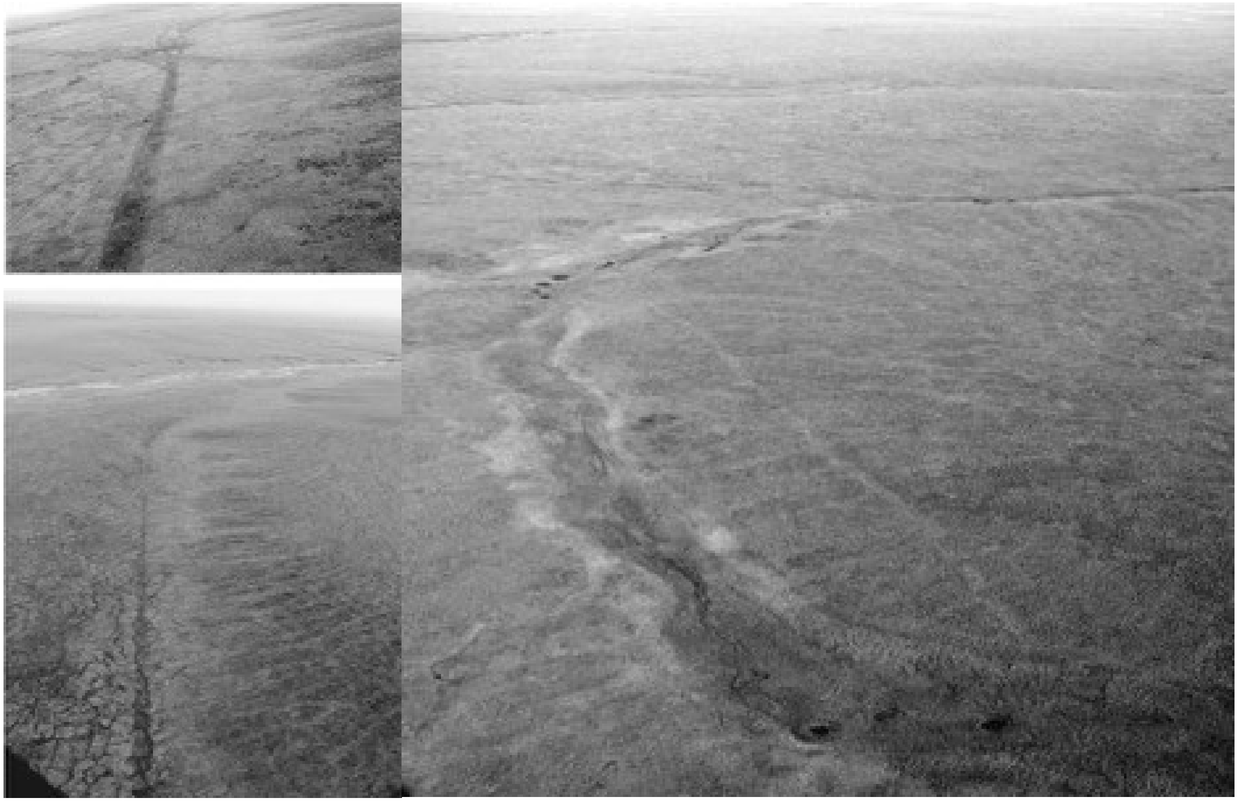

Figure S15. Trails in the 1002 Area of the Arctic National Wildlife Refuge made by camp-move vehicles during 2D seismic surveys in 1984 and 1985. The top-left image was taken in July 1985 of a trail through ice-rich permafrost terrain; the lower-left image is of the same trail taken in July 2007. An undisturbed reference plot to the left of the trail had a soil excess ice content of 28% in 1985. Thawing of soil ice and ice wedges led to trail subsidence. The trail remained wetter and greener than surrounding tundra in 2007. The right image shows a trail created in 1984 and photographed in 2005. The trail was still visible after 21 years because it had fewer evergreen shrubs, more sedge and flatter microtopography than the surrounding tundra (Jorgenson et al. 2010). (Photos: U.S. Fish & Wildlife Service)

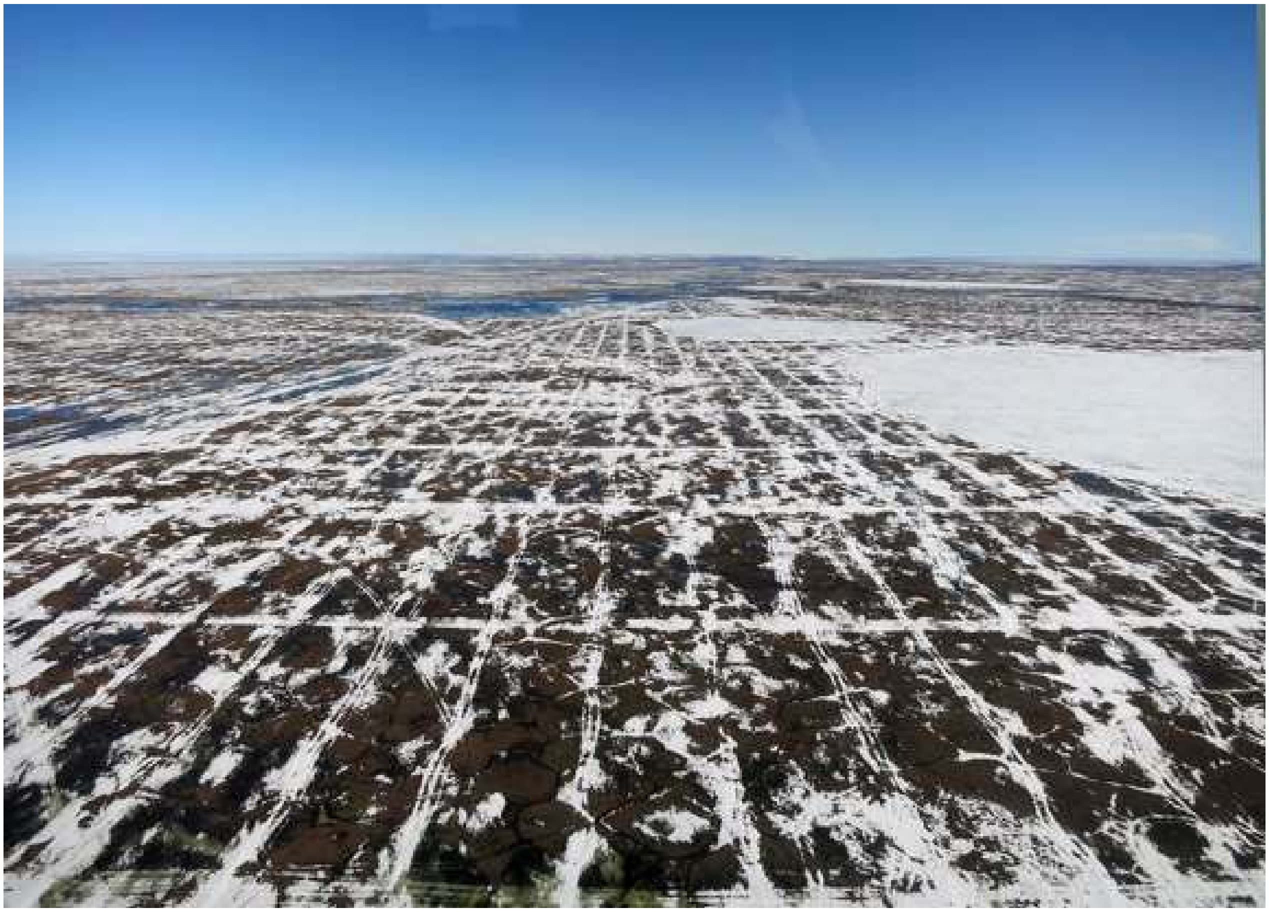

Figure S16. Trails left by the 3D Icewine seismic survey in spring 2018, approximately 40 km south of the Prudhoe Bay oilfield and 20 km west of the Dalton Highway. The survey consisted of seismic lines spaced 37.5 m to 150 m apart and covered approximately 518 km^2 (Alaska Department of Natural Resources 2018). (Photo: H. Buelow)

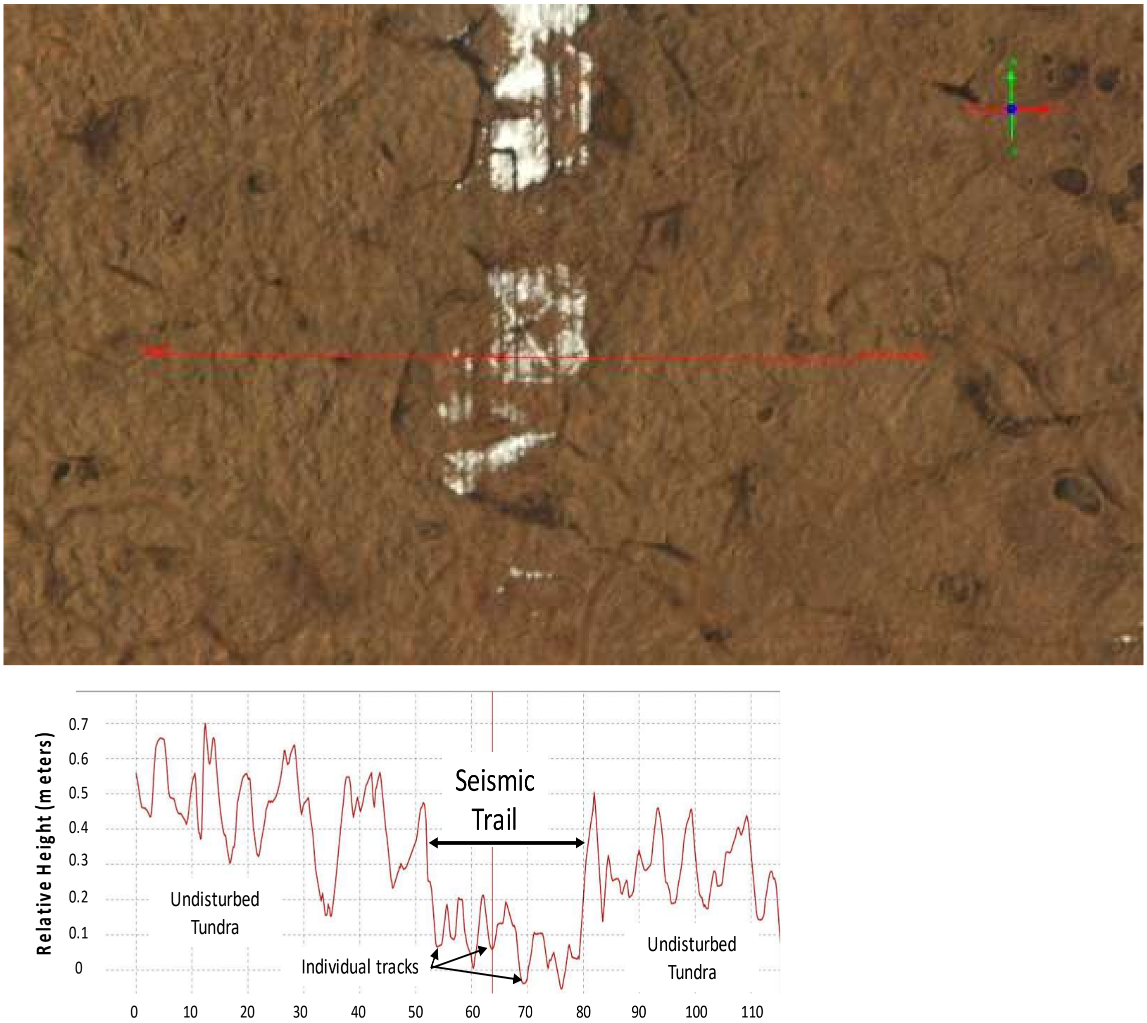

Figure S17. An airborne photogrammetric analysis of compression of tundra surface in one of the seismic lines from a 2018 3D-seismic survey near the Canning River, just west of the Arctic National Wildlife Refuge. The upper aerial orthophoto shows a corridor of vehicle trails with compacted late-melting snow and tracks left by numerous tracked vehicles. The horizontal red line denotes a 115-m digital topographic transect used to extract elevations from the digital elevation model created to study these impacts. The transect includes approximately 50 m of undisturbed tundra on the left side (gentle hill) and 35 m on the right side (flatter terrain) of the 30-m wide seismic line. The red dot denotes the center of the seismic line at approximately 63 m along the transect. The lower chart shows the elevations along the 115-m transect. The vertical (y) axis is microtopographic variation (0.1-m intervals), and the horizontal axis depicts distance along the transect (10-m intervals). The vertical red line corresponds to the approximate center of the seismic line at the red dot. There was 20–40 cm of topographic variability associated with moss hummocks, tussocks, and ice-wedge polygon rims and troughs in the undisturbed tundra on both sides of the seismic line. Within the seismic trail, the tundra was compressed approximately 20 cm below the adjacent level on either side of the trail, had half the microrelief, and approximately 10–25 cm ruts from individual vehicle tracks (unpublished data, M. Nolan 2018).

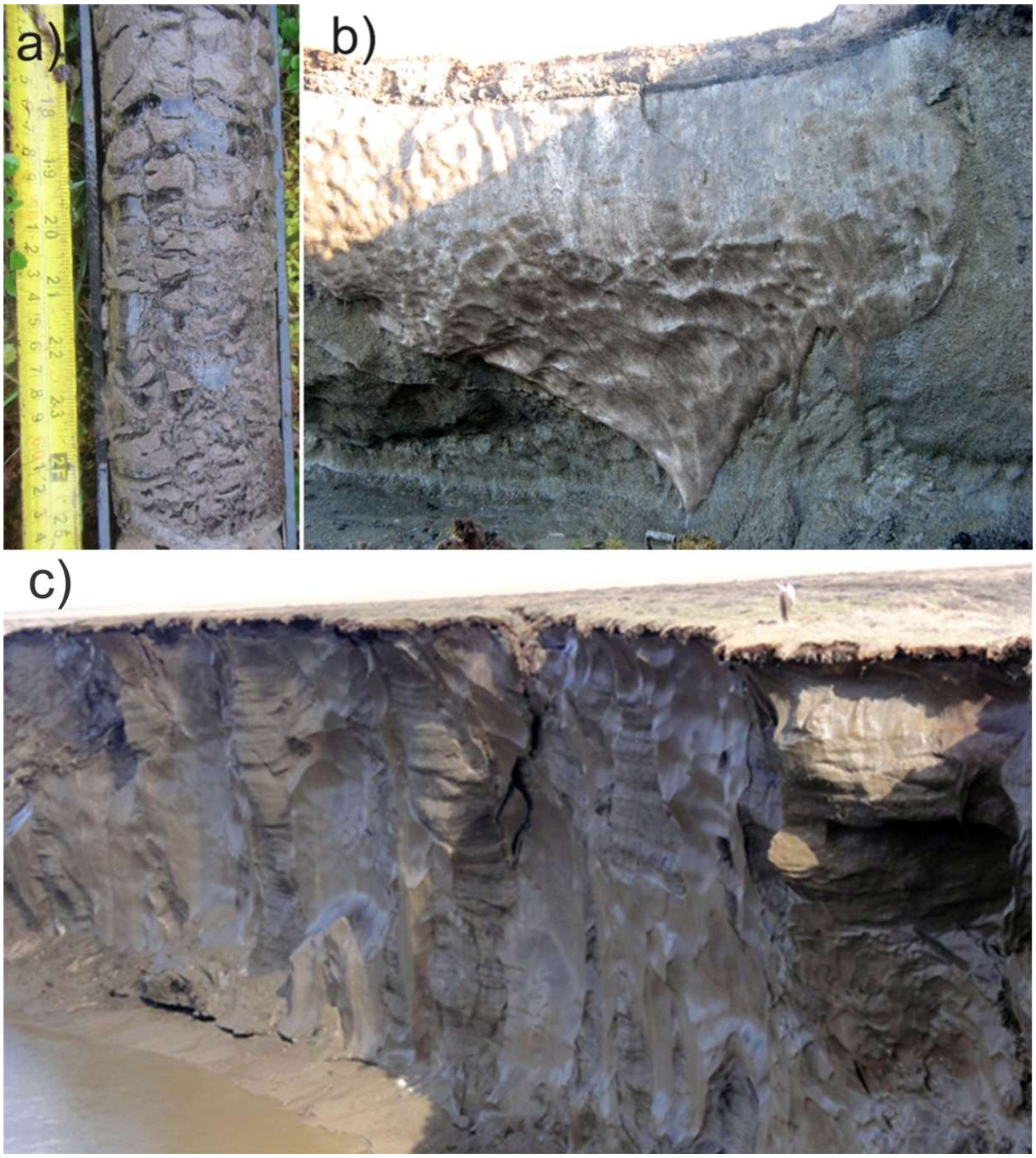

Figure S18. (a) Ice-rich permafrost in core, which was over 80% ice by volume. (b) Ice wedge at the Beaufort Sea coast, northern Alaska. This ice is one of the most common forms of massive ground ice in permafrost, and is responsible for the prominent ice-wedge polygons visible in aerial photographs of the region (e.g. Figure S19 and S20). This ice wedge was approximately 4 m deep and over 5 m wide at the top. (c) A bluff along the Itkillik River Exposure, showing the massive ice in the yedoma deposit (Kanevskiy et al. 2011). For scale, note person standing at top of bluff. (Photos: M. Kanevskiy)

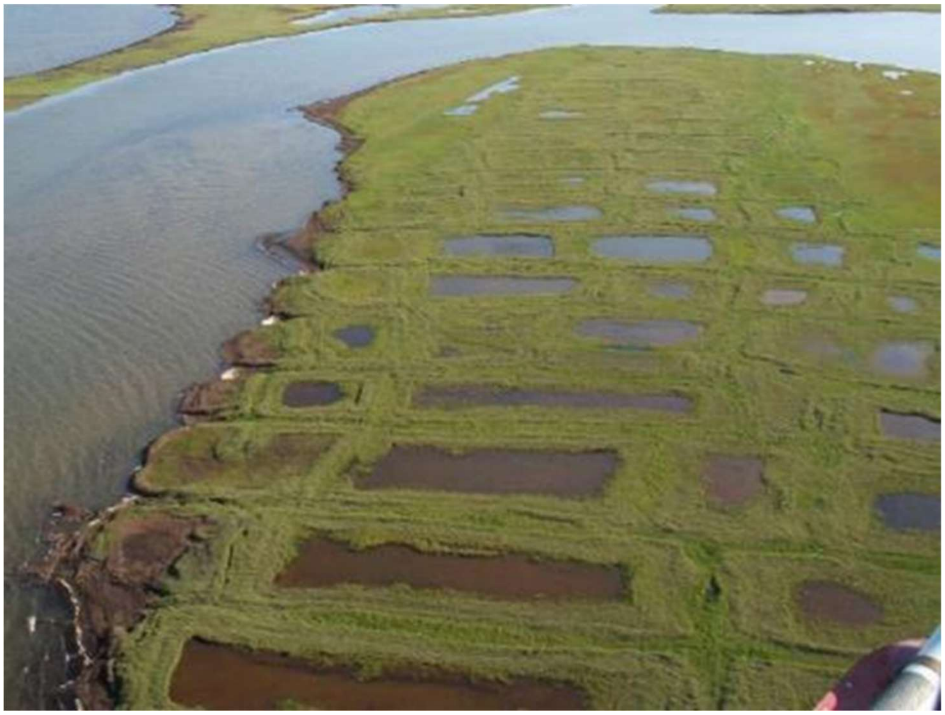

Figure S19. Low-centered polygons with intra-polygonal ponds associated with ice-wedge development, Arctic Coastal Plain, northern Alaska. (Photo: M. Kanevskiy)

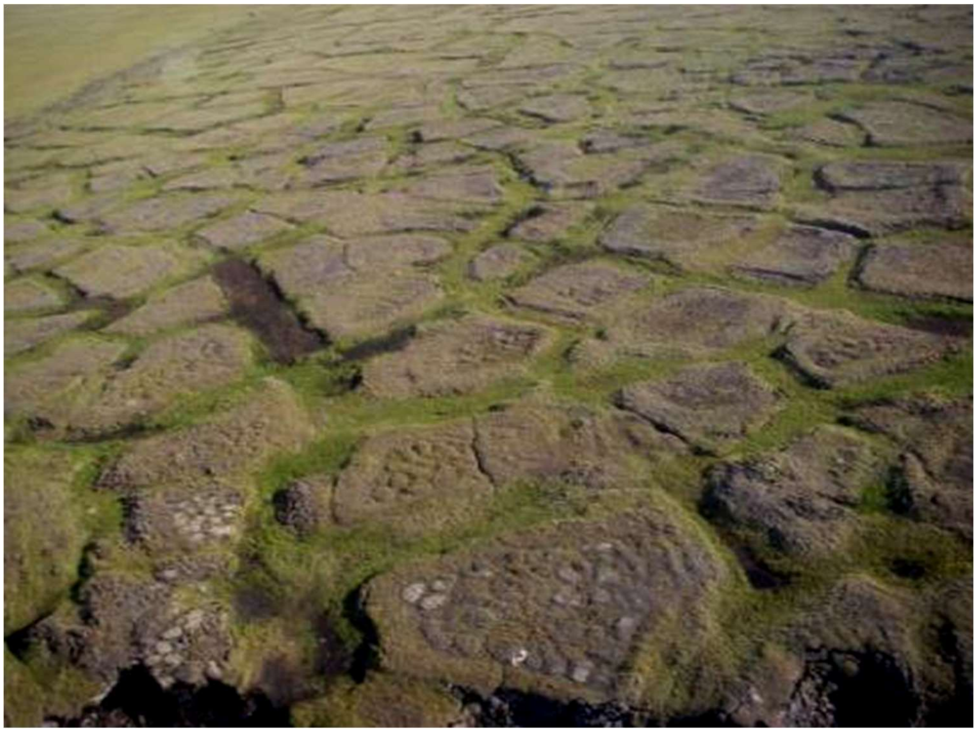

Figure S20. High-centered polygons with troughs over partially degraded ice wedges, Arctic Coastal Plain, northern Alaska. (Photo: M. Kanevskiy)

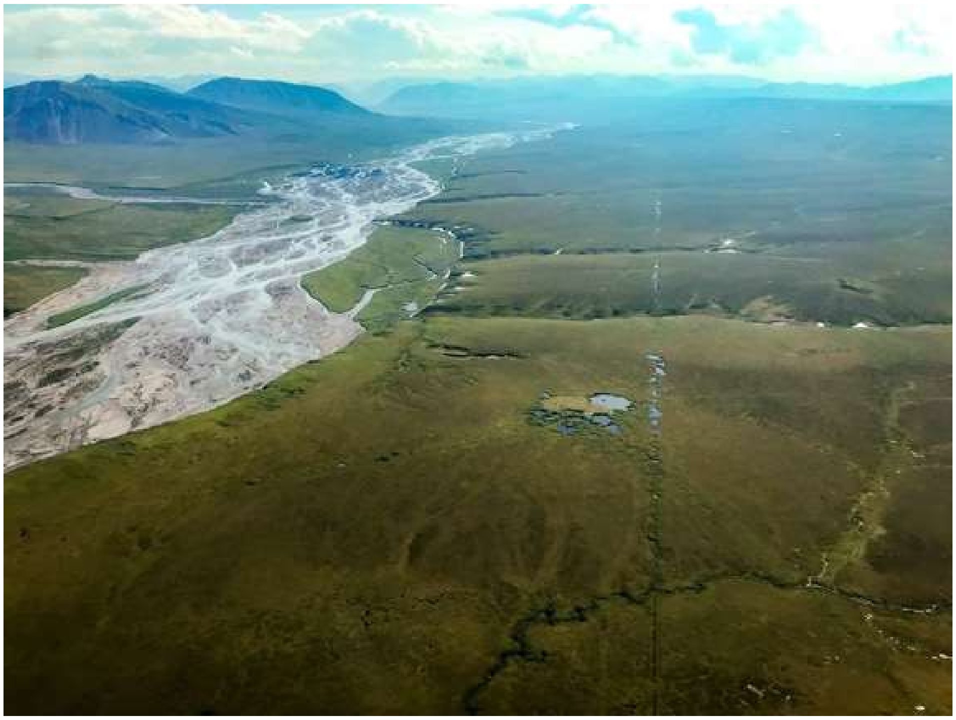

Figure S21. One of many bladed tractor trails just west of the Canning River and the Arctic National Wildlife Refuge, caused by seismic exploration in the 1960s, photographed in 2018. Note the extensive thermokarst and ponding of water along the trail and around the gravel drilling pad near the center of the photo, abandoned since 1974. (Photo: M. Nolan, Fairbanks Fodar, *http://fairbanksfodar.com/happy-10-02-day.* Accessed 28 July 2018)

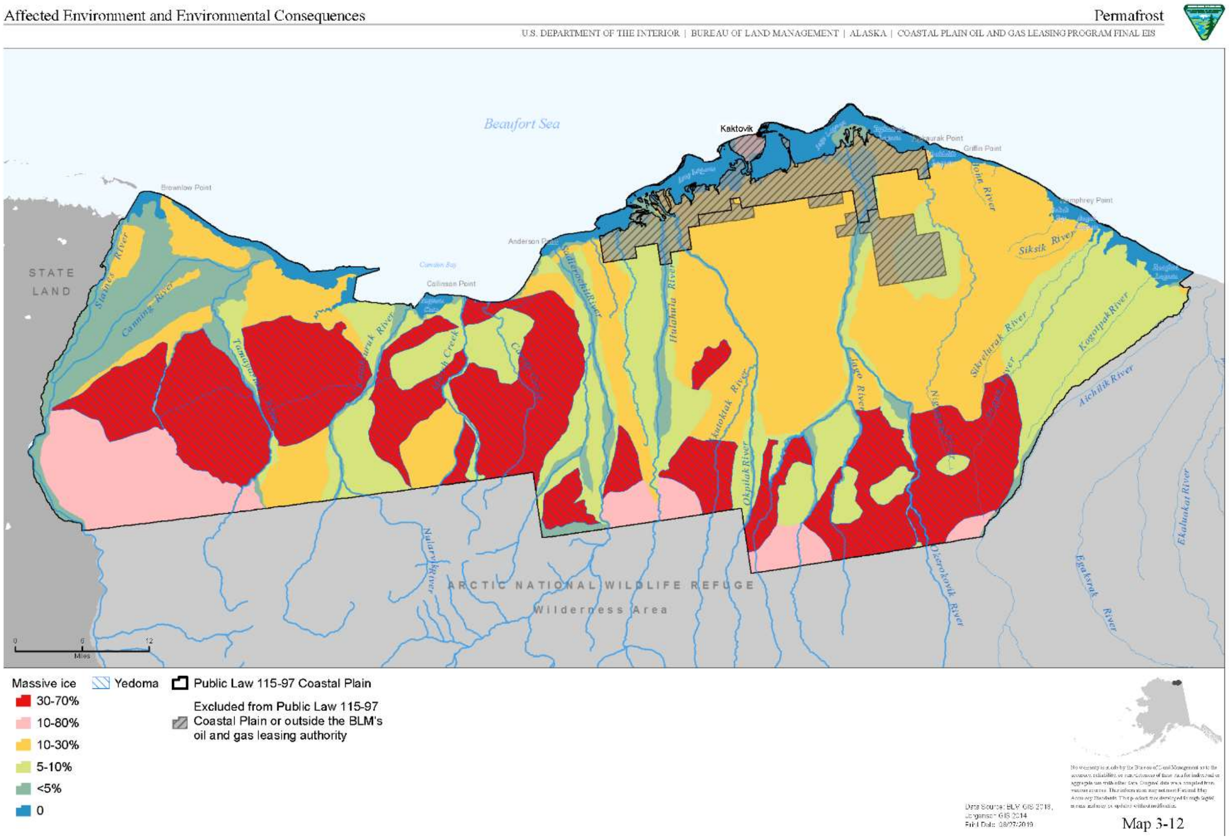

Figure S22. Map of ice-rich permafrost from the Coastal Plain Oil and Gas Leasing Program Environmental Impact Statement, showing the approximate extent of yedoma with massive ice (red color with diagonal blue stripes) (Map 3-12, Bureau of Land Management 2019, based on Jorgenson et al 2014).

References

Alaska Department of Natural Resources. 2018. MLUPNS 17-002, Geokinetics Inc., Icewine 2018 3D, Geophysical Exploration Permit Approval. (Letter from Alaska Department of Natural Resources). . Retrieved 11 Nov 2018 from *http://dog.dnr.alaska.gov/Documents/Permitting/NorthSlope/MLUP/* 2018/MLUPNS_17-002_Geokinetics_Icewine_2018_3D_GeophysicalExplorationPermitApproval.pdf.

Bureau of Land Management. 2019. Coastal Plain Oil and Gas Leasing Program Environmental Impact Statement. https://eplanning.blm.gov/epl-front-office/projects/nepa/102555/20003762/250004418/Volume_1_ExecSummary_Ch1-3_References_Glossary.pdf. Accessed 12 Sept 2019.

Jones, B. M., R. Rykhus, Z. Lu, C. D. Arp, and D. J. Selkowitz. 2008. Radar imaging of winter seismic survey activity in the National Petroleum Reserve-Alaska. Polar Record **44**:227–231.

Jorgenson, J. C. 2018. Tundra disturbance and recovery on winter seismic trails in the Arctic National Wildlife Refuge, monitored from 1985 to 2018. Arctic National Wildlife Refuge, U.S. Fish and Wildlife Service, Fairbanks, Alaska, US.

Jorgenson, J. C., J. M. V. Hoef, and M. T. Jorgenson. 2010. Long-term recovery patterns of arctic tundra after winter seismic exploration. Ecological Applications **20**:205-221.

Jorgenson, M. T., M. Kanevskiy, Y. Shur, J. Grunblatt, C.-L. Ping, and G. Michaelson. 2014. Permafrost database development, characterization, and mapping for northern Alaska for the Arctic Landscape Conservation Cooperative. Alaska Ecoscience and University of Alaska Fairbanks.

Jorgenson, J. C., B. E. Reitz, and M. K. Raynolds. 1996. Tundra disturbance and recovery nine years after winter seismic exploration in northern Alaska. Arctic National Wildlife Refuge, U.S. Fish and Wildlife Service, Anchorage, Alaska.

Kanevskiy, M., Y. Shur, D. Fortier, M. T. Jorgenson, and E. Stephani. 2011. Cryostratigraphy of late Pleistocene syngenetic permafrost (yedoma) in northern Alaska, Itkillik River exposure. Quaternary Research **75**:584-596.

Nolan, M. 2018. Detecting tire tracks in the 1002 Area with Fodar. *http://fairbanksfodar.com/detecting-tire-tracks-in-the-1002-area-with-fodar*.

Raynolds, M. K., and N. A. Felix. 1989. Airphoto analysis of winter seismic trails in northeastern Alaska. Arctic **42**:362-367.

SAExploration. 2018. Marsh Creek 3D plan of operations winter seismic survey. Retrieved from *https://eplanning.blm.gov/epl-front-office/projects/nepa/111085/153349/187888/Marsh_Creek_Plan_* of_Operations_Submitted_May2018.pdf.

van Everdingen, R., editor. 2005. Multi-language glossary of permafrost and related ground-ice terms. National Snow and Ice Data Center/World Data Center for Glaciology, Boulder, CO.
